# Role of Photobiomodulation Therapy in Neurological Primary Burning Mouth Syndrome. A Systematic Review and Meta-Analysis of Human Randomised Controlled Clinical Trials

**DOI:** 10.3390/pharmaceutics13111838

**Published:** 2021-11-02

**Authors:** Reem Hanna, Snehal Dalvi, Rene Jean Bensadoun, Judith E. Raber-Durlacher, Stefano Benedicenti

**Affiliations:** 1 Laser Therapy Centre, Department of Surgical Sciences and Integrated Diagnostics, University of Genoa, Viale Benedetto XV, 6, 16132 Genoa, Italy; stefano.benedicenti@unige.it; 2 Department of Oral Surgery, Dental Institute, King’s College Hospital NHS Foundation Trust, London SE5 9RS, UK; 3 Department of Periodontology, Swargiya Dadasaheb Kalmegh Smruti Dental College and Hospital, Nagpur 441110, India; drsnehaldeotale@gmail.com; 4 Centre De Haute Energie, Department of Oncology Radiology, 10 Boulevard Pasteur, 06000 Nice, France; renejean.bensadoun@che-nice.com; 5 Academic Centre for Dentistry Amsterdam, Department of Oral Medicine, University of Amsterdam, Gustav Mahlerlaan 3004, 1081 LA Amsterdam, The Netherlands; judith@raber.nl; 6 Department of Oral and Maxillofacial Surgery, Amsterdam UMC, University of Amsterdam, De Boelelaan 1117, 1118, 1081 HV Amsterdam, The Netherlands

**Keywords:** oxidative stress, trigeminal nerve inflammation, neuropathic pain, primary burning mouth syndrome, mitochondrial homeostasis, photobiomodulation, transmucosal, molecular mechanisms, RCT, outcome measures

## Abstract

Mitochondrial homeostasis is crucial for energy production and neuronal survival in neurological primary burning mouth syndrome (npBMS). Photobiomodulation therapy (PBMT) has been utilised in npBMS management, however, its role of intervention remains controversial. The aim of this systematic review and meta-analysis of CRD 42020198921 PROSPERO registration reference was to oversee and determine the efficacy of PBMT in patients with npBMS, identifying the gaps and bridge them by proposing recommendations for future studies purposes. PRISMA guidelines and Cochrane Collaboration recommendations followed. Various search engines employed to analyse a total of 351 studies of which 12 were included. A wide range of utilised PBM wavelengths was between 635–980 nm and the power output ranged between 30 mW and 4000 mW. A high risk of bias (RoB) was noted in 7 out of 12 included studies (58.3%), as results of qualitative analysis. Meta-analysis findings of 4 out of 12 studies showed statistically significant intergroup differences (SSID) for visual analogue scale (VAS) values (MD = −1.47; 95% CI = −2.40 to −0.53; Z = 3.07 (*p* = 0.002) whereas meta-analysis on 5 out of 12 studies revealed SSID for anxiety/depression and quality of life (MD = −1.47; 95% CI = −2.40 to −0.53; Z = 3.07 (*p* = 0.002), favouring PBMT group to the control treatment strategies. Despite the inconsistency and diversity in PBM parameters (wavelength, power, light source, spot size, emission mode, energy per point, total energy) and treatment protocols (exposure time, number of sessions, time interval between sessions, treatment duration)—majority of the included studies showed positive PBM results. The high RoB and meta-analytical heterogeneity in the eligible studies warrant the necessity to perform well-designed and robust RCTs after acknowledging the drawbacks of the available scientific literature and addressing our suggested recommendations highlighted in our review.

## Highlights

1. Neurological primary burning mouth syndrome (npBMS) has significant functional and psychological impacts on patient’s quality of life.
2. Despite the positive results of the included studies in this review favouring photobiomodulation therapy in npBMS, high RoB and heterogeneity.
3. The high RoB and meta-analytical heterogeneity in the eligible studies warrant the necessity to perform well-designed and robust RCTs, taking into consideration the drawbacks and the gaps of these studies and utilise our proposed suggested recommendations to bridge the gaps.

## 1. Introduction

Idiopathic or primary burning mouth syndrome (BMS) is defined by the International Headache Society (IHS) as “an intraoral burning or dysaesthetic sensation, recurring daily for more than two hours per day over more than three months, without evident causative lesions on clinical examination and investigation” [1]. In addition to the oral burning or stinging sensation of the tongue, lips or other oral mucosal surfaces, patients with BMS frequently report dry mouth (xerostomia), taste disturbance (dysgeusia) and tingling or paraesthetic sensations [1]. BMS depends on its clinical features and its response to therapies. It is also a term of “stomatodynia” referred to persistent idiopathic orofacial pain based on their homogenous topography feature according to the available multivariate analysis [2]. Despite many studies that have utilised various BMS diagnostic criteria, their findings remain controversial due to limitations of some BMS classification criteria. A review conducted by Mosisset et al. (2016) [3] showed that none of the included studies before 2014 have used the latest IHS criteria 2013 [4]. Additionally, authors using International Association for the Study of Pain (IASP) criteria [5] can include heterogeneous patients, some of whom present with burning sensations that do not fulfil current BMS criteria [6]. This can indicate a diversity in the clinical outcomes.

Diagnosis and classification of BMS based on the presented clinical symptoms is significant in paving the way for the appropriate treatment, to achieve the optimal outcomes. The taste thresholds within both the fungiform and foliate papillae are impaired in patients with BMS [7], leading to gustatory disturbances such as: dysgeusia and parageusia [7,8]. Ultimately, BMS and trigeminal neuropathic pain have a significant impact on the patient’s quality of life (QoL) [9].

**Figure 1:**
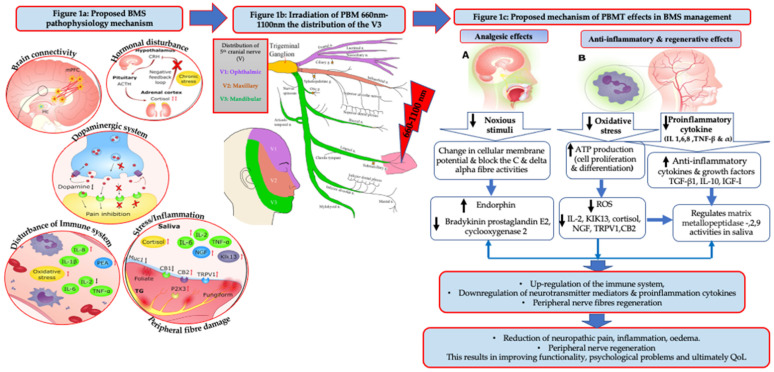
(**a**–**c**). Schematic representation of the proposed BMS pathophysiology mechanism (**a**) and PBM-irradiation of the tongue (main target) where it shows the irradiation of the V3 distributions (**b**) and proposed mechanism of action of PBM in BMS management (**c**). In Figure 1c, “A“ represents the analgesic effects of PBMT whereas “B” represents the anti-inflammatory and regenerative effects of PBMT. Abbreviations: BMS: burning mouth syndrome; IL: Interleukin; TNF-β and α: transforming necrosis factor-beta and alpha; NGF: nerve growth factor; TRPV-1: transient receptor potential cation channel subfamily V member 1; ROS: reactive oxygen species; ATP: adenosine triphosphate; MMP-1,2,9: matrix metalloproteinases-1,2,9; PBM: photobiomodulation; nm: nanometre; V3: mandibular branch of the 5th cranial nerve (trigeminal nerve).

The pathogenesis of BMS is complex, multifactorial and remains uncertain. However, oxidative stress (OS) has been associated with several diseases, such as neurodegenerative disorders [10] and anxiety BMS [11,12] (Figure 1). The mitochondrial homeostatic mechanism is vital for energy production and neuronal regeneration under stress conditions and is closely modulated by mitochondrial biogenesis and selective mitochondrial degradation [13]. However, several neurological dysfunctions and neurodegenerative conditions can contribute to mitochondrial dysfunction [14], leading to a lack of adenosine triphosphate (ATP), resulting in Na^+^/K^+^ ATPase failure and in primary afferent neurons malfunction. This can participate in abnormal characteristic of neuropathic pain (NP) activity [15]. Moreover, an increase in reactive oxygen species (ROS) and ground plasmic calcium (Ca^+2^) imbalances and mechanisms have previously associated with NP pathogenesis [16,17]. Additionally, many in vivo animal studies showed significant lower density of epithelial nerve fibres in oral mucosa [18] and a lower number of fibres penetration of the epithelium in the oral mucosa of patients with BMS [19]. These changes were observed in the peripheral nervous system suggesting BMS to be a small fibre trigeminal neuropathic condition [20]. This was supported by immunohistochemical studies which have demonstrated a significant loss of epithelial and subepithelial nerve fibres [19,21] together with an increased expression of nerve growth factor (NGF) [22], transient receptor potential cation channel subfamily V member 1 (TRPV1) ion channels, as well as cannabinoid receptor type 2 (CB2) in modulating P2X3 receptors in the primary afferent neuron of tongue mucosa of BMS patients and associated with some changes in trigeminal reflexes [20,23,24].

It is noteworthy that despite the fact that anxiety and depression are reported in patients with BMS, such conditions commonly arise only after BMS onset [25]. Moreover, much evidence links BMS with lesions and/or dysfunction in the central and peripheral nervous systems [26] and gustatory nociceptive [27].

The pharmacotherapeutics (capsaicin, alpha-lipoic acid, benzodiazepines, benzylamine hydrochloride, selective serotonin re-uptake inhibitors (SSRIs) and tricyclic antidepressants) of BMS are based on antagonising the neuropathic signalling pathways, which as highlighted above alleviate the neuropathic pain [28,29]. This could be utilised as a single or combined with non-pharmacotherapy (cognitive behavioural measures) [30]. It is noteworthy that some systematic medications are effective in the short-term, but can be associated with major side effects, threatening its large scale and long-term use [31]. There is evidence of patients who have discontinued long-term opioid therapy (especially oral opioids) due to adverse events or insufficient pain relief; however, weak evidence suggests that patients who are able to continue opioids long-term, experience clinically significant pain alleviation and inconclusive QoL and functional improvement [32]. A systematic review and meta-analysis conducted by Sommet et al. (2015) showed that in short-term studies (4–12 weeks) of chronic neuropathic pain, opioids were superior to placebo in terms of efficacy and inferior in terms of tolerability, however, opioids and placebo did not differ in terms of safety [33]. Despite the many randomised controlled trials (RCT) studies of BMS therapeutic interventions that have been examined in many reviews, a lack of consistency in their results has been reported, due to their methodology, sample size and relatively short follow-up timepoints or a lack of medications targeting specific receptors at both the peripheral and central targets of trigeminal and gustatory fibres [34,35]. Ultimately, there is little research evidence that provides a clear guidance for clinicians to treat those patients and for scholars to take the research forward.

Based on the above notes, photobiomodulation (PBM) therapy (PBMT) has emerged, as a possible alternative to standard care treatments. Prior to 2014, PBMT used to be called low-level laser therapy (LLLT), which both referring to the same therapy. The cellular and molecular mechanisms of PBMT suggest that the photonic energy of red and near-infrared (NIR) [36] are absorbed by cytochrome c oxidase (CCO), which is the mitochondrial chromophore situation on its cell membrane [37], resulting in cellular respiration upregulation, an increase in mitochondrial membrane potential and ATP production [38], ROS modulation [39], a release of nitric oxide (NO) [40], and subsequently a release of intracellular Ca^+2^ [41]. Various transcript factors activation and signalling pathways stimulation, resulting in an increase in cell proliferation and production of; antioxidant, anti-inflammatory, proangiogenic factors and anti-apoptotic activities [42,43]. PBM modulates inflammatory and oxidative signalling pathways relevant in BMS pathophysiology, including NF-κB and MAPK signalling. Additionally, PBM alters the nerve conduction and excitation in peripheral neurons by its action on the Na+/K+ pump [44], resulting in noxious stimuli reduction, through its effects on selective TRPV1 and NGF signalling blockers, decreasing their expressions (blockage of inflammatory thermal hyperalgesia) [45] (Figure 1). The above notes have great practical benefits in pain alleviation, peripheral nerve regeneration, functionality improvement, thereby, patients’ psychological parameters and QoL can be enhanced.

PBMT has been utilised in orofacial pain management [46,47], including BMS [48,49,50,51]. Despite many clinical studies investigating PBMT efficacy and effectiveness in BMS management, a diversity in their findings remain a challenge for reproducibility. This has been well-demonstrated in a recent systematic review and meta-analysis, which have focused on PBMT effectiveness or efficacy in patients with BMS [52,53,54,55], concluding that further robust RCTs and methodology are warranted, due to the heterogeneity in the results of their included RCTs and a lack of standardised PBM parameters.

The rationale of conducting our systematic review and meta-analysis was to examine the reasons of the discrepancy/or inconsistency in the results of the available clinical RCTs studies and attempt to provide suggested recommendations for standardised methodology and PBM parameters to guide scholars and investigators to conduct further extensive research in neurological primary BMS management. Hence, the present systematic review and meta-analysis is aimed to appraise and underpin the prevailing scientific evidence, justifying the gaps and drawing up a substantial structure to reach unequivocal efficacy of PBMT in neurological primary BMS. The objectives of this research review are listed below:

1. To investigate the core of the inconsistencies among the available data and extrapolate the reasons.
2. To evaluate the sensitivity of the results’ methods of assessment and obtain vigorous standardised methodology, taking into consideration the appropriate diagnostic criteria.
3. To attempt to propose a preliminary empirical consensus of PBM dosimetry and treatment protocols.
4. To postulate extraoral and intraoral treatment strategies for BMS for future clinical RCTs.

## 2. Materials and Methods

### 2.1. Review Protocol and PROSPERO Registration

This systematic review was carried out according to the guidelines of the Preferred Reporting Items for Systematic Reviews and Meta-Analysis (PRISMA) statement and Cochrane Handbook for Systematic Reviews (Supplementary File S1) [56,57]. Review protocol is registered in Prospective Register of Systematic Reviews (PROSPERO); ref CRD 42020198921.

### 2.2. Population (P), Intervention (I), Comparison (C) and Outcomes (O): PICO

**P**: Subjects diagnosed with neurological primary BMS according to any criteria.
**I**: Utilisation of PBMT; laser-PBM or light-emitted diode (LED)-PBM, as a mono-therapy or combined.
**C**: Placebo (Sham PBM) or pharmacotherapy (topical or systematic), or cognitive approach or physiotherapy.
**O**: Evaluation of patient’s self-reporting outcomes (pain intensity including burning sensation, functional problems, anxiety/depression, QoL), immunohistochemistry and salivary profiles.

### 2.3. Focused Questions of Review Search

This systematic and meta-analysis was based on the PICO strategy, in order to answer the following focused research review questions:

1. Does PBM with laser or LED or combined therapies have superior effects compared to placebo or any primary BMS standard care, in reducing neuropathic pain intensity, improving patients’ functionality, psychological status and QoL?
2. Does combined laser-PBM therapy of red or NIR wavelengths prove synergistic effects compared to placebo?
3. Do the diagnostic criteria of primary neurological BMS play a role in optimising the clinical outcome of patients with BMS?
4. Is it possible to propose clinical guidance and recommendations of PBMT (LED and laser) for BMS management?

### 2.4. Search Strategy

The search strategy was carried out by two review authors (R.H. and S.D.) independently, including only terms related to, or describing, the study domain and intervention. With the view to evaluate the inter-reviewer reliability, Kappa (κ) statistics were performed with a minimum value of 0.8 deemed to be acceptable [58]. In the event of any inconsistency or disagreement, a third review author (S.B.) was asked to solve the matter. The following databases, using the relevant keywords and Medical Subjective Headings (MeSH) Terms, were systematically searched: Cochrane Library database, MEDLINE (NCBI PubMed and PMC), EMBASE, CINAHL, ClinicalTrials.gov, ProQuest, Scopus, RCTs Registry Trial, comparing PMBT with a placebo or any standard care intervention or combined therapies in patients with primary neurological BMS, Cochrane Central Register of Controlled Trials (CCRCT), ScienceDirect and Google Scholar.

Additionally, the following journals were manually searched: *Photomedicine and Laser Surgery*, *Journal of Headache and Pain*, *Cephalalgia*, *Journal of Dental Research*, *Lasers in Medical Science*, *Journal of Photochemistry and Photobiology*, *Pain Journal*, *Journal of Orofacial Pain*, *Medicine*, *J. Phys. Therapy Sci.*, *BMJ Open*, *J. Craniofac. Surg.*, *Journal of Neuroscience*, *Nature Neuroscience*, *J. Clin. Exp. Dent*, *Med. Oral Patol. Oral Cir. Bucal*, *Journal of Oral Rehabilitation*, *J. Craniomandibular Disord.*, *Clin. J. Pain*, *Laser Ther.* and *Journal of Biophotonics*. The electronic search was thoroughly explored during the period 1 January 2010—28 February 2021.

### 2.5. Relevant Free Keywords and MeSH Terms

The resources Medical Subject Headings (MeSH), Health Sciences Descriptors (DeCS) and Embase Subject Headings were used to select the search descriptors as well relevant free keywords. The Boolean operators “AND” and “OR” were used to improve the search strategy through various combinations. The following terms were searched in combination:

“Burning mouth syndrome” **OR** “burning tongue” **OR** “Oral burning” **OR** “glossalgia” **OR** “glossodynia” **OR** “glossopyros” **OR** “stomatodynia” **OR** “stomatopyros” **OR** “dysaesthesia” **OR** “stomatodynia” **OR** “stomatopyrosis” **OR** “glossopyrosis” **OR** “sore mouth” **OR** “sore tongue” **OR** “oral dysesthesia” **OR “BMS”**

**AND**

“Low-level laser therapy” **OR** “LLLT” **OR** “laser” **OR** “photobiomodulation” **OR** “Light” **OR** “infrared” **OR** “monochromatic” **OR** “NIR” **OR** “near infrared” **OR** “phototherapy” **OR** “laser” **OR** “photobiomodulation” **OR** “PBM” **OR** “LLLT” **OR** “low power laser therapy” **OR** “Biostimulation” **OR** “light emitted diodes” OR “LEDs”

**AND**

“Randomised controlled trials” **OR** “RCT”

### 2.6. Eligibility Criteria

#### 2.6.1. Inclusion Criteria

1. Subjects of both genders aged ≥18-year-old diagnosed with neurological primary burning mouth syndrome (npBMS), according to any orofacial neuropathic pain diagnostic criteria.
2. Randomised clinical trials (RCTs) with no period restriction, published in any language dealt with the evaluation of the effectiveness of PBMT in the treatment of primary neurological BMS symptoms, compared to placebo (PBM sham) or any standard care treatment.
3. Symptoms’ duration without intraoral lesions ≥3 months.
4. Subjects with no physiological or systematic conditions, contributing into the pain.
5. RCT’s comparing the efficacy of PBMT to any other standard treatment modality.
6. All in vivo human RCTs’ designs.
7. No wavelengths restrictions that are within the optical window regardless of the light source, whether laser or LED.
8. No restrictions on the reported laser and LED parameters.
9. Studies reporting at least one of the following parameters, as an outcome variable: Pain, burning sensation, functionality problems, QoL, anxiety/depression, salivary flow profile, immunohistochemistry biomarkers.
10. RCT studies with the longest follow up of at least 1 month after treatment.
11. Search engine period from 1 January 2010–28 February 2021.

#### 2.6.2. Exclusion Criteria

1. In vitro and in vivo animal studies, case reports, letter to the editor and/or editorials, literature review, systematic review/or meta-analysis, books and book chapters, pilot study and indexes and abstracts or university work assignment with insufficient data (letters, personal opinions, conference abstracts).
2. Studies with subjects who were on antidepressant, anxiolytic, or anticonvulsant drugs <3 months.
3. Subjects who underwent chemo- and/or radiotherapy.
4. Studies utilised PBMT and medication, as a primary intervention.
5. Hyposalivation related to Sjogren syndrome (unstimulated saliva production ≤ 0.1 mL/min) or any predisposing factors not related to BMS.
6. Subjects with secondary burning mouth syndrome.
7. Pregnant and lactating women.
8. Intraoral mucosal lesions.
9. Subject with the following neuropathic orofacial pain: trigeminal neuralgia, glossopharyngeal neuralgia, oral Iatrogenic pain, primary burning mouth syndrome, temporomandibular joint dysfunction syndrome, migraine, odonatological and head and neck origins.
10. Systematic diseases/or on medications induce neuropathic pain.
11. BMS patient has been treated previously phototherapy.
12. Patients unable to follow the indications for administration of oral topical medications
13. Subjects with pain related to bone conditions.
14. Subjects with any of the following: neurological disorders metabolic disorders, autoimmune disorders, diabetic mellitus.
15. Subjects with parafunctional habits or intra-oral trauma or local nerve damage.
16. Studies used PBM-acupuncture with or without medications, as a primary intervention.

### 2.7. Types of Outcome Measure

#### 2.7.1. Primary Outcome

Changes in the pain intensity level/or intraoral burning sensation from baseline up to the end of the follow-up timepoints. Table 1 shows the qualitative (patient-reported outcomes subjective) and quantitative measures (objective) utilised in the eligible studies.

#### 2.7.2. Secondary Outcome

Changes in the below outcomes from baseline up to the end of the follow-up timepoints:

1. Functionality problems: dysgeusia (taste), sleepiness.
2. QoL/overall treatment improvement.
3. Anxiety/depression.
4. Immunohistochemistry profile.
5. Salivary flow profile.
6. Any reported adverse effect.

### 2.8. Qualitative Analysis

A qualitative assessment for each study was carried out, using the Revised Cochrane Risk-of-Bias (RoB) tool for Randomised trials, Version 2.0 (RoB 2), by two independent reviewers [R.H. and S.D.] [59,60]. Detailed assessment under the following headings was performed:

1. Bias arising from the randomization process;
2. Bias due to deviations from intended interventions;
3. Bias due to missing outcome data;
4. Bias in measurement of the outcome;
5. Bias in selection of the reported result.

Depending upon fulfilment of above-mentioned criteria, the eligible studies were determined as “low”, “moderate” or “high” RoB. Disagreements between the reviewers were resolved by discussion with a third author (S.B.) as well as, use of ‘discrepancy check’ feature in RoB 2, in order to obtain consensus.

### 2.9. Statistical Analysis of Data

The RevMan v5.4.1 was utilised to carry out a random effects meta-analysis for continuous outcomes of the data of interest extracted from the included studies in this review [61]. The random effects model was chosen to evaluate the presence of heterogeneity, if any amongst the eligible studies. Pertinent numerical data on the primary outcome measure (pain reduction assessment by qualitative measurement with VAS) and secondary outcome measure (anxiety/depression and QoL assessment by qualitative measurement with OHIP), was exported from the chosen studies from the baseline evaluation up to the final follow-up evaluation. The pooled standardised mean differences (SMDs) with associated 95% confidence intervals (95% CIs) were used to calculate the treatment effects and the pooled overall effect was considered statistically significant when *p* < 0.05 [62]. As a means to identify statistical heterogeneity, visual inspection of forest plots was conducted and outlier studies, if any, were identified [62]. *I*^2^ statistics for homogeneity ranged from 0 to 100% with the following interpretation: 0% = no evidence of heterogeneity; 30–60% = moderate heterogeneity; and 75–100% = high heterogeneity [63]. In the event, visual assessment of funnel plot symmetry was utilised to detect the presence of publication bias [64].

## 3. Results

### 3.1. Study Selection

**Figure 2:**
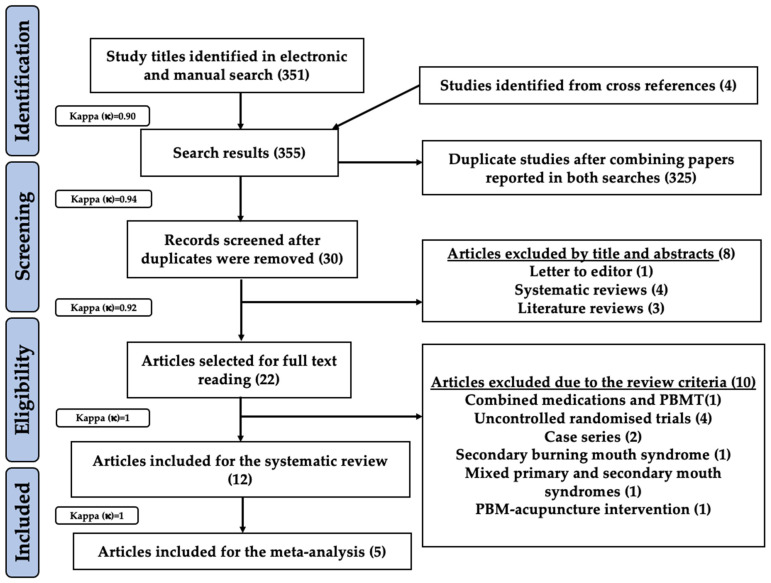
PRISMA flow-chart of selected criteria for the eligible articles.

In total, 351 study titles were shortlisted after a thorough combined electronic and manual search for possible eligibility in this systematic review and meta-analysis. Additionally, four study titles were obtained from cross-references. Hence, preliminary screening revealed a total of 355 eligible study titles (inter-reviewer agreement, κ = 0.90). After combining papers reported in both searches, 325 duplicate studies were excluded resulting in further evaluation of 30 records (inter-reviewer agreement, κ = 0.94). Subsequently, the following eight articles were excluded based on their titles and abstracts: one (letter to editor), four (systematic reviews) and three (literature reviews) (inter-reviewer agreement, κ = 0.92). Thus, 22 study articles were scrutinized based on our eligibility criteria. Ten studies were excluded for the following reasons; combined medications and PBMT [65]; uncontrolled randomised trials [47,66,67,68]; case series [69,70]; secondary BMS [71]; mixed primary and secondary BMS [72]; PBM-acupuncture intervention [73] (inter-reviewer agreement, κ = 1). Therefore, 12 studies were qualified for the present systematic review [74,75,76,77,78,79,80,81,82,83,84,85] and five studies qualified for a meta-analysis [74,75,79,82,84] (inter-reviewer agreement, κ = 1). The search strategy utilised in the present systematic review and meta-analysis has been illustrated in the PRISMA flow diagram (Figure 2). 

We analysed the results of the eligible studies based on their pBMS diagnostic criteria, as follows: studies utilised IASP diagnostic criteria revised 2013 [86], studies utilised IASP diagnostic criteria 2016 [87], studies utilised ICHD-3-diagnostic criteria, 2nd Edition (2013) [4] and studies utilised ICHD-3-diagnostic criteria, 3rd edition, 2018 [1], which are illustrated in Section 3.2, Section 3.3, Section 3.4 and Section 3.5 respectively.

### 3.2. Studies Utilised IASP Diagnostic Criteria Revised 2013 

#### 3.2.1. Characteristics of the Study Populations

In total, 2 out of 12 studies utilised IASP diagnostic criteria revised 2013 [78,81]. The sample size distribution amongst these studies was as follows; *n* = 30 (7 lost to follow-up) in one study [78] and *n* = 40 in the other study [81]. With regards to the age distribution, one study reported the mean age of 59.7-year-old (yrs) [78], while in the other study the mean age in PBM group was 60.2 yrs and in placebo group was 61.1 yrs [81].

In terms of gender distribution, one study recruited only females [78], whereas one study was conducted on more than a 50% female population [81]. Any relevant data on patients’ racial background in the included studies were reported and classified as follows: Black, Black/Caucasian, non-Caucasian for the purpose of this systematic review and meta-analysis. Both studies failed to report the patients’ racial background [78,81]. Both studies reported a combination of presented symptoms; burning sensation and dysgeusia [78]; burning sensation and pain [81]. The duration of presented symptoms was not specified in both the studies [78,81], whereas the affected areas were reported in one study as follows: tongue (T), upper lip (UL), lower lip (LL), buccal mucosa (BM), mandibular ridge (MR), soft palate (SP), hard palate (HP) and lower gingivae (LG) [78] and the other study failed to provide this information [81]. Likewise, one study mentioned xerostomia and dysgeusia, as functionality problems [78], while the other study failed to provide this information [81]. Table 2 refers to the characteristics of the study populations amongst the eligible studies.

#### 3.2.2. Study Characteristics

One of the two studies in this cohort was conducted in Brazil [78] while the other was conducted in Croatia [81]. The authors of one study have mentioned that their study was single-blind (SB) RCT [78], while the other study was an RCT, but the authors failed to mention the blinding details [81]. In both studies, PBMT was compared to placebo (sham) group [78,81]. Table 2 refers to the study characteristics amongst the eligible studies.

#### 3.2.3. Documentation of Reported PBM Irradiation Parameters

The wavelength utilised in one study was 790 nm diode laser [78], while 680 nm (type of device unspecified) was utilised in the other study [81]. Both studies utilised a continuous emission mode (CW) in their respective studies and additionally they failed to provide any information on laser tip-tissue distance (contact/non-contact mode) [78,81]. In terms of reported energy and energy density, one study utilised 6 J/point and 6 J/cm^2^, respectively [78]. Whereas, the other study has not reported energy, but mentioned the energy density of 3 J/cm^2^ [81]. In terms of the power output and irradiance, one study utilised 120 mW (0.12 W) and 4 W/cm^2^, respectively [78], while the other study reported power output of 30 mW, but the irradiance was unspecified [81]. The exposure time was 50 s/point [78] and 100 sec/point [81], respectively.

The treatment frequency, time interval and duration of treatment was twice a week for two weeks in one study [78], while the other study did not mention this information [81]. In terms of spot size/spot area and beam diameter/fibre-tip diameter parameters, one study utilised 0.03 cm^2^ [78], while the other study employed 2 mm, 1 cm^2^ surface area [81].

Method of PBM applications and number and allocation of trigger points (TP) in the two studies were as follows; 24 sites for PBMT group (T, LL, UL, BM, MR, palate (P), LG) and 17 sites for control group (T, LL, UL, BM, MR, P, LG) [78]; tongue mucosa, number and allocation of TP: no relevant information [81]. Table 3 refers to the laser parameters utilised amongst the eligible studies.

#### 3.2.4. Assessment Methods

Both studies have assessed pain intensity [78,81], but one of them has additionally assessed unstimulated whole salivary flow (UWS) [81]. Pain assessment was carried out by VAS assessment for both studies [78,81]. Additionally, one study used the global perception chart of pain index to evaluate pain intensity [78], whereas the other study evaluated the immunohistochemistry profile by determining the salivary levels of tumour necrosis factor-alpha (TNF-α) and interleukin (IL)-6 levels using ELISA [81]. Table 1 refers to the various assessment methods which were utilised amongst the eligible studies.

### 3.3. Studies Utilised IASP Diagnostic Criteria 2016 

#### 3.3.1. Characteristics of the Study Populations

Six out of 12 studies utilized the IASP diagnostic criteria 2016 [74,76,77,79,80,82]. Sample size distribution in the studies was as follows; *n* = 20–25 [76,80], *n* = 30–35 [77], *n* = 40–45 [82], *n* = 75–85 [74,79]. Age distribution in the studies was as follows; 45–54 yrs. [76], 55–64 yrs. [74,79,80], 65–74 yrs. [77], range 56–83 yrs. with mean age: 67.56 yrs. [82]. Four studies included more than 50% females [77,79,80,82], while one study each included equal numbers of male and female patients [74] and only female patients [76], respectively. Only one study reported the patients’ racial background as Caucasian [77], whereas all other studies in this cohort failed to report the relevant data [74,76,79,80,82].

The distribution of presented symptoms was as follows; five studies reported burning sensation [74,76,77,79,80], four studies reported pain [74,76,77,79] and one study reported burning mouth symptoms [82]. Duration of presented symptoms was reported as follows; >3 months [80], 4 months [76], >6 months [74], 6 months [77,79], no relevant information [82].

The distribution of affected areas was as follows; tip tongue (TT), lateral tongue (LT), dorsal tongue (DT), UL, LL, BM [74], TT, DT, LT (bilateral), UL, LL, HP, SP [79], ten areas of oral mucosa: BM, T, floor of mouth, (FM) SP, HP [76], oral mucosa [77], both sites: tongue, lip or HP [80], no relevant information [82].

In terms of functionality problems, two studies reported functional limitation, physical pain, psychological and social disabilities [74,77], one study reported taste disturbance, pain intensity [76], one study reported xerostomia, intraoral (IO) disability [80], while two studies failed to report the relevant information [79,82]. Table 2 refers to the characteristics of the study populations amongst the eligible studies.

#### 3.3.2. Study Characteristics

The distribution of studies based on their country of origin was as follows; two studies each in Italy [74,77] and Croatia [80,82] and one study each in Iran [76] and Spain [79]. Three studies conducted double-blind (DB) RCT [74,79,80], two studies conducted single-blind (SB) RCT [76,82] whereas one study failed to provide any relevant data [77]. The intervention groups in the studies were as follows; PBMT versus (vs) placebo [74,76,80,82], PBMT vs medication [77], multiple PBMT wavelengths vs Placebo [79]. Table 2 refers to the study characteristics amongst the eligible studies.

#### 3.3.3. Documentation of Reported PBM Irradiation Parameters

The wavelength utilised in this cohort has been documented as follows; 660–970 nm [74], diode laser 630 nm [76], Diode laser 980 nm [77], groups 1 and 2 (G1 and 2): IR-laser 830 nm, group 3 (G3): Red-laser 635 nm [79], Ga-Al-As LED 685 nm [80], GaAlAs laser 830 nm [82].

Two studies utilised a CW emission mode [77,79]. One study each utilised; gated mode: 800 ms on/1 ms off, 80% duty cycle [82], pulsed mode—50% [74], pulsed mode [80]. One study failed to provide any relevant information on the emission mode [76].

The laser tip-tissue distance (contact/non-contact) was reported as follows; 2 mm (non-contact) [77], 0.5 cm (non-contact) [80], 5 mm (non-contact) [82], no relevant information [74,76,79]. Reported energy was 1 J in one study [76], G1 and G2: IR-laser 830 nm, G3: Red-laser 635 nm in one study [79] while four studies provided no information [74,77,80,82].

The fluence was reported as follows; 1 J/cm^2^/area [76], 10 J/cm^2^ [77], G1 and G2: 176 J/cm^2^ G3: 72 J/cm^2^ [79], 2 J/cm^2^ (Total 60 J/cm^2^) [80], 12 J/cm^2^ [82] and no relevant information [74].

The power output across the studies was; 30 mW [76,80], 300 mW [77], 3200 mW [74], G1 and G2: 100 mW, G3: 35 mW [79], 100 mW (Average) [82].

In terms of the irradiance the following information was obtained; 1 W/cm^2^ [77], G1 and G2: 3.57 W/cm^2^, G3:1.25 W/cm^2^ [79], 0.003 W/cm^2^ [80], no relevant information in three studies [74,76,80]. The exposure time was reported as follows; 3 min and 51 s [74], 10 s [76], 10 s/point [77], G1 and G2: 50 s and G3: 58 s [79], 381 s/point [80] and 5 min/session [82].

In terms of treatment frequency, time interval and duration of treatment a vast heterogeneity in the data was noted which has been recorded as follows; once a week for 10 weeks [74]; twice a week for 4 weeks [76]; twice a week for 5 weeks (total of 10 sessions) [77]; G1 (830 nm): one session/week for 10 weeks, G2 (830 nm): three sessions/week for 9 weeks, G3 (635 nm): three sessions/week for 9 weeks, control group (CG): three sessions/week for 9 weeks [79]; daily for 10 days excluding weekend [80]; once per day excluding weekend for 4 weeks (total of 10 sessions) [82].

The spot size/spot area/beam diameter/fibre-tip diameter parameters were as follows; two studies reported 1 cm^2^ [74,82], one study each reported prob diameter of 0.28 cm^2^ and spot size of 0.6 cm [77], whereas one study reported only the spot size of 3 cm^2^ [80]. However, two studies failed to provide any relevant information [76,79].

A noticeable heterogeneity was noted amongst the studies in this cohort for methods of PBM applications, number and allocation of TP which have been reported as follows; IO, TP- no information [74]; Total 10 TP (TP/site): T:2, floor mouth (FM):2, SP:1 and HP:1 [76]; IO: apex tongue (AT):3, LT:4, DT:10, BM:8, labial mucosa (LAM): 5, HP:8, SP:3, gingivae (G) and alveolar ridge mucosa (ARM): three each [79]; three reported burning sites (no information on number and location) [80]. Two studies failed to provide any relevant information on these parameters [77,82]. Table 3 refers to the laser parameters and protocols utilised amongst the eligible studies.

#### 3.3.4. Assessment Methods

The following assessment methods were carried out in this cohort; pain assessment in five out of six studies [74,76,77,79,82], QoL assessment in four out of six studies [76,77,79,82], stress/anxiety/depression assessment in two studies [74,77] and one study each evaluated functionality limitations [74], physical activity [74], PH saliva [77] and salivary cortisol level [80]. Furthermore, the following are the evaluation methods utilised in the eligible. studies of this review: VAS assessment in five out of six studies [74,77,79,80,82]. In terms of Oral Health Impacts Profile (OHIP) assessment, five out of six studies assessed this parameter [74,76,77,79,82]. However, different versions of the questionnaire were utilised in the studies; Italian-OHIP [74], Persian-OHIP [76], OHIP-49 [77], OHIP-14 [79], OHIP-CRO-14 (Croatian) [82]. One study each utilised numerical rating scale (NRS) [76], McGill questionnaire (MPQ) [77], present pain intensity (PPI) scale [77], hospital anxiety-depression scale (HADS) [77], geriatric depression scale (GDS) [77], UWS pH [77], visual numerical scale (VNS) [79], unstimulated saliva (ELISA) [80]. Table 1 refers to the various assessment methods which were utilised amongst the eligible studies.

### 3.4. Studies Utilised ICHD-3-Diagnostic Criteria, 2nd Edition (2013)

#### 3.4.1. Characteristics of the Study Populations

In total, 2 out of 12 studies were included in the cohort which utilised the ICHD-3-diagnostic criteria, 2nd edition (2013) [75,83]. The sample size was *n* = 55 in one study [75] and *n* = 21 in the other study [83]. Both studies included a patient population with more than 50% females who were in the age range 65–70 yrs. [75,83]. Both studies failed to report the patients’ racial background [75,83].

The presented symptoms and their duration were categorized as follows; oral burning/pain for a duration ≥ 6 months [75], pain/burning, anxiety/depression for three months [83]. While the affected areas were mentioned in one study as TT, LT, DT, BM, LM, HP, SP, G, alveolar mucosa (AM) [83], the other study failed to provide this information [75]. In terms of functionality problems, one study reported pain, oral burning sensation, reduction in saliva flow [75] and the other reported IO and psychological disabilities [83]. Table 2 refers to the characteristics of the study populations amongst the eligible studies.

#### 3.4.2. Study Characteristics

Both studies included in this cohort were conducted in Spain [75,83]. One of them was a prospective, partially blinded, single centre RCT [75], whereas the other study was a DB-RCT [83]. Additionally, in both studies, PBMT was compared to sham PBM [75,83]. Table 2 refers to the study characteristics amongst the eligible studies.

#### 3.4.3. Documentation of Reported PBM Irradiation Parameters

In terms of the wavelength, both studies [75,83] utilised GaAIAs diode laser 808–815 nm. The CW emission mode was utilised; however, laser tip-tissue distance (contact/non-contact) was unspecified [75,83].

The energy parameter in two studies was reported as follows; LLLT group (G1): 4 J/point, LLLT group (G2): 6 J/point [75]; 3 J/point [83], whereas the fluence was recorded as follows; G1:133.3, G2:200 J/cm^2^ [75] but no relevant information reported by Spanemberg et al. (2019) [83].

With regards to the power output, one study utilised 1 W [75] and the other study utilised 200 mW [83]. In terms of irradiance, one study utilisation 1.97 W/cm^2^ [83], while the other study provided no information [75]. The following data were reported for exposure time; G1 (4 J/point): 4 s/point, G2 (6 J/point): 6 s/point [75]; 15 s/point [83].

In terms of the treatment frequency, time interval and duration of treatment, one study reported the following information; G1: once a week, G2: six times a week and duration of treatment for G1and G2 was 4 weeks [75]; whereas, the other study reported utilisation of eight sessions in total, twice a week for 4 weeks [83].

Spot size/spot area/beam diameter/fibre-tip diameter parameters were reported as follows; 0.03 cm^2^ [75]; 0.088 cm^2^ [83]. One study reported methods of PBM applications, number and allocation of TP, as IO TP: 10 [75]; while the other study reported total: 41 (bilateral) TP/site: TT: 3, LT: 4, DT: 10, BM: 8, LAM: 5, HP: 8, SP: 3, G or AM: 3 [83]. Table 2 refers to the laser parameters and protocols utilised amongst the eligible studies.

#### 3.4.4. Assessment Methods

The assessment methods in the two studies were reported as follows; pain, oral health, salivary flow, anxiety/depression, over all treatment satisfaction in one study [75], pain/burning sensation, dry mouth, dysphagia in the other study [83]. In terms of the evaluation methods, both studies utilised the VAS assessment [75,83]. One study additionally utilised OHIP-14 (Spanish version), Sialometry, HADS, patient global impression of improvement (PGI-I) [75] while the other study utilised HADS [83]. Table 1 refers to the various assessment methods which were utilised amongst the eligible studies.

### 3.5. Studies Utilised ICHD-3-Diagnostic Criteria, 3rd Edition, 2018 

#### 3.5.1. Characteristics of the Study Populations

The ICHD-3-diagnostic criteria, 3rd edition, 2018 was utilised in one out of 12 studies included in this systematic review and meta-analysis [84]. The sample size for this study was *n* = 20 patients [84]. This study included more than 50% female population in the age group between 65 and 70 yrs. [84], however, it failed to report the patients’ racial background. The presented symptoms in this study were pain/burning sensation, depression/anxiety and lack of sleep for a duration of <3 months [84]. The affected areas were vestibular mucosa (VM), lip (L), BM, HP, LT, DT, sublingual (S) [84]. The patients reported functionality problems such as: IO disability, mental and psychological disabilities and lack of sleep [84]. Table 2 refers to the characteristics of the study populations amongst the eligible studies.

#### 3.5.2. Study Characteristics

This study was a SB-RCT compared PBMT with placebo and was conducted in Spain [84]. Table 2 refers to the study characteristics amongst the eligible studies.

#### 3.5.3. Documentation of Reported PBM Irradiation Parameters

The PBM irradiation parameters reported in this study were; utilised wavelength 810 nm diode laser, CW emission mode, 2 mm laser tip-tissue distance (non-contact), energy—6 J/point, fluence—12 J/cm^2^, power output—0.6 W, irradiance—1.2 W/cm^2^, exposure time—10 s/point [84]. PBM therapy was applied twice a week for five weeks (10 sessions in total) [84]. The spot size/area was reported as 0.5 cm^2^ and the beam diameter was 300 μ [84] (Table 3). Method of PBM application was IO: 56 points VM: 3 (4 sites), LM: 4, bilateral BM: 6/site, HP: 6, bilateral LT: 4/site, DT: 6, S: 4 bilateral [84]. Table 3 refers to the laser parameters and protocols utilised amongst the eligible studies.

#### 3.5.4. Assessment Methods

The parameters assessed in this study were pain, sleepiness, QoL, anxiety/depression [84]. These parameters were assessed by the following evaluation methods; VAS, short form-36 health survey questionnaires (SF-36), psychometric symptoms checklist-90-R (SCL90-R), McGill-questionnaire [84]. Table 1 refers to the various assessment methods which were utilised amongst the eligible studies.

### 3.6. Studies Utilised Unspecified Criteria

#### 3.6.1. Characteristics of the Study Populations

In total, 1 study out of the 12 eligible studies did not specify the criteria for BMS assessment [85]. This study was performed on 40 female patients with a mean age of 62.06 ± 3.1 years who reported burning sensation (duration of symptoms unspecified) [85]. The study failed to report the patients’ racial background [85]. The affected areas were reported as; upper labial mucosa (ULM), DT, BM, lower labial mucosa (LLM) and the functionality problem reported was pain [85]. Table 2 represents the characteristics of the study populations amongst the eligible studies.

#### 3.6.2. Study Characteristics

This study was a DB-RCT which compared PBMT vs placebo and was conducted in Spain [85]. Table 2 refers to the study characteristics amongst the eligible studies.

#### 3.6.3. Documentation of Reported PBM Irradiation Parameters

PBM irradiation parameters reported in this study were documented as follows; utilized wavelength diode LED 805 nm, laser tip-tissue distance (contact/non-contact)—4 cm spacer used (non-contact), energy—1200 J (total), fluence—50 J/cm^2^, power output—4 W (total), irradiance—166.7 mW/cm^2^, exposure time—300 s/area, twice a week PBM irradiation for four weeks (eight sessions in total) [85].

In terms of methods of PBM applications, the following are the obtained information related to number and allocation of TP; IO points: four areas (BM, LAM, DT, LLM), number of TP unspecified [85]. Additionally, the authors of this study have failed to provide information on the emission mode and the spot size/spot area/beam diameter/fibre-tip diameter parameters [85]. Table 3 represents the utilized laser parameters and protocols amongst the eligible studies.

#### 3.6.4. Assessment Methods

The following parameters were assessed in this study: pain and capillary bed of the target tissue in terms of: length, diameter, density and morphology tortuosity [85]. All the parameters were assessed by the following evaluation methods: VAS, NRS, video-capillaroscopy evaluation [85]. Table 1 refers to the various assessment methods which were utilised amongst the eligible studies.

### 3.7. Qualitative Assessment

**Figure 3:**
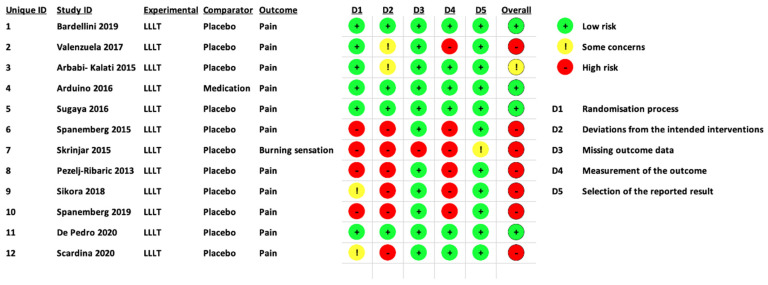
Risk of bias assessment summary of the included studies based on the consensual answers of two individual assessors (R.H. and S.D.).

**Figure 4:**
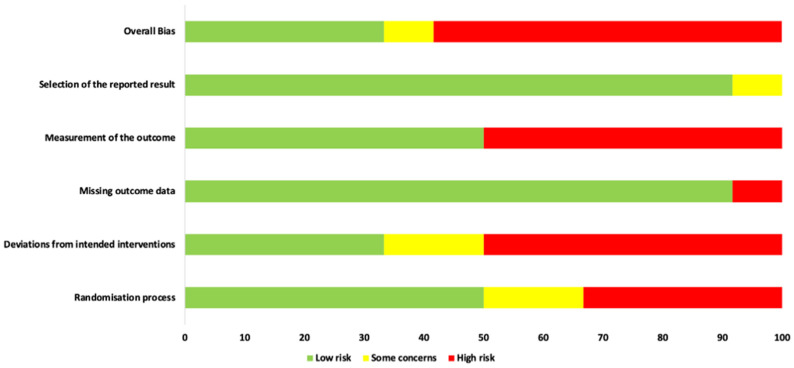
Risk of bias assessment graph of the included studies expressed as percentages based on the consensual answers of two individual assessors (R.H. and S.D.).

The RoB 2 tool that is designed for in vivo human RCTs was utilised to assess all the selected studies for their quality, as shown in Figure 3 and Figure 4 [57,60,88]. Figure 3 shows RoB assessment summary of all the eligible studies, whereas Figure 4 is a domain-wise graphical representation of RoB score percentage evaluated using this tool. Both figures represent the consensual answers verified using the “discrepancy check” feature of the RoB 2 tool, across two independent review authors (R.H. and S.D.) (inter-reviewer agreement, κ = 0.94). Fifty percent (six studies) of the included studies were at low risk of inadequate randomisation [74,75,76,77,78,84], whereas 16.7% (two studies) [82,85] and 33.3% (four studies) [79,80,81,83] studies had some concerns or were at high risk, respectively.

Amongst the included studies, 33.33% studies were at a low risk of deviations from the intended interventions (four studies) [74,77,78,84], while 16.7% (two studies) [75,76] and 50% (six studies) [79,80,81,82,83,85] were at a high risk, respectively. In terms of missing outcome data, 91.7% studies were at a low risk of bias (11 studies) [74,75,76,77,78,79,81,82,83,84,85] whereas 8.3% of the included studies (one study) [80] were at a high risk of bias, respectively. Fifty percent (six studies) [74,76,77,78,84,85] of the studies were at a low risk for measurement of outcome whereas the remaining 50% studies (six studies) [75,79,80,81,82,83] were at a high risk of bias.

In terms of the selection of the reported results, a low risk of bias was reported in 91.7% studies (11 studies) [74,75,76,77,78,79,81,82,83,84,85], whereas 8.3% of the included studies (one study) [80] showed some concerns, respectively. The overall risk of bias assessment revealed that 33.3% of the included studies (4 studies) [74,77,78,84] were at a low risk of bias, 8.3% (one study) [76] and 58.3% of the included studies (seven studies) [75,79,80,81,82,83,85] were at a high risk of bias.

### 3.8. Impact Factor of the Published Papers

In total, 4 out of 12 studies were published in high-impact-factor (IF) journals of “>2” [75,79,83,84]. A total of 5 out of 12 studies were published in moderate-IF “between 1 and 2” journals [74,76,77,78,81]. In total, 2 out of 12 studies were published in low-IF “<1” journals [80,82]. The journal of one study failed to specify its impact factor, which might imply a low impact factor [85]. It is noteworthy that published papers in journals of various impact factors could be a reflection of convenience rather than a set of absolute values. Table 2 refers to the impact factor of the eligible studies.

### 3.9. Quantitative Assessment

#### Outcome Variables

The treatment outcomes were broadly based into primary and secondary outcomes which were assessed by qualitative and quantitative measures as illustrated in Table 3. The primary outcomes for this review were pain/burning sensation reduction, which were further subdivided as patient reported/qualitative (subjective) outcomes such as: VAS, NSP, PPI and quantitative (objective) outcomes such as MPQ. The secondary outcome variables were classified under three categories namely, functional improvement, anxiety/depression and QoL and over all treatment satisfaction. Functional improvement methods included salivary analysis profile (quantitatively assessed by sialometry (UWS pH), TNF-α and IL-6 levels ELISA (Unstimulated saliva)), microcirculation assessment [quantitatively assessed by video capillaroscopy evaluating the capillary bed: parametric data (capillary loop length, diameter, density and tortuosity) and non-parametric data (presence of capillaries with particular morphology)], immunohistochemistry analysis (quantitatively assessed by IL-8, IL-1β, IL-6, IL-2, TNF-α). Anxiety/depression and QoL were assessed under the following categories: patient self-reported/qualitative (subjective) outcomes such as: BAI, PAD, HRQL, OHIP-14 (all versions) and quantitative (objective) outcomes such as: HADS, SCL-90-R, EuroQol-5D-5L, GDS, SF-36. Overall treatment satisfaction was assessed quantitatively by PGI-I.

Table 4 describes the level of significance between PBMT and the control group for different outcome variables enlisted in Table 1, amongst the included studies in this systematic review and meta-analysis. A total of 8 out of 12 studies reported statistically significant results for qualitative assessment of pain/burning sensation reduction [74,75,76,77,78,79,83,84], whereas four studies reported non-significant statistical results [80,81,82,85].

Quantitative assessment of pain/burning sensation reduction was performed in only 2 out of 12 studies [77,84] where statistically significant results were reported. Functional improvement was quantitatively assessed in 5 out of 12 studies of which four studies reported non-significant statistical results [75,80,81,85] and one study reported statistically significant results [77]. Anxiety, depression and QoL were qualitatively assessed in 6 out of 12 studies of which five studies reported statistically significant results [74,75,76,79,84] and one study reported non-significant statistical results [82]. Quantitative assessment of anxiety depression and QoL was performed in 4 out of 12 studies of which three studies reported statistically significant results [77,83,84] and one study reported insignificant statistical results [75]. Furthermore, quantitative assessment of overall treatment satisfaction was performed in 2 out of 12 studies of which one study reported statistically significant results [78], whereas one study reported insignificant statistical results [75].

**Figure 5:**
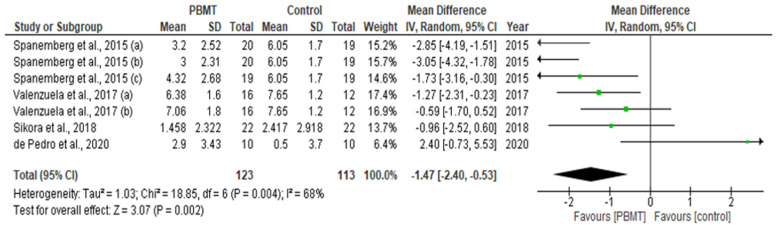
Forest plot for primary outcome qualitative pain/burning sensation reduction assessment (VAS) from baseline up to the final follow-up timepoint.

**Figure 6:**
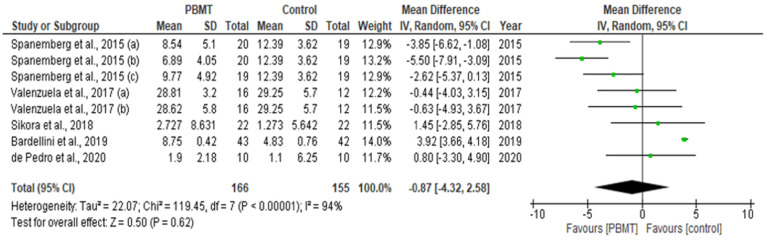
Forest plot for secondary outcome qualitative anxiety/depression and QoL assessment (OHIP) from baseline up to the final follow-up timepoint.

Out of 12 eligible studies, four studies with relevant numerical data for the primary outcome measure (pain reduction assessment by qualitative measurement with VAS) contributed to this meta-analysis [75,79,82,84]. Data extracted from a total of 236 patients, evaluated from baseline up to the final follow-up evaluation for each study, were pooled to reveal a statistically significant inter-group difference (MD = −1.47; 95% CI = −2.40 to −0.53; Z = 3.07 (*p* = 0.002), favouring the PBMT group, along with a substantial high heterogeneity (T^2^ = 1.03; X^2^ = 18.85; df = 6; (*p* = 0.004); I^2^ = 68%) amongst the included studies (Figure 5). In total, 5 out of 12 eligible studies with relevant numerical data for the secondary outcome measure (anxiety/depression and QoL assessment by qualitative measurement with OHIP) contributed to this meta-analysis [74,75,79,82,84]. Data extracted from a total of 321 patients, evaluated from baseline up to the final follow-up evaluation for each study, were pooled to reveal a statistically significant inter-group difference (MD = −1.47; 95% CI = −2.40 to −0.53; Z = 3.07 (*p* = 0.002), favouring the PBMT group, along with a substantial high heterogeneity (T^2^ = 22.07; X^2^ = 119.45; df = 7; (*p <* 0.0001); I^2^ = 94%) amongst the included studies (Figure 6). A meta-analysis on other outcome variables could not be conducted owing to the disparity in methodology and incomplete or incomparable numerical data.

**Figure 7:**
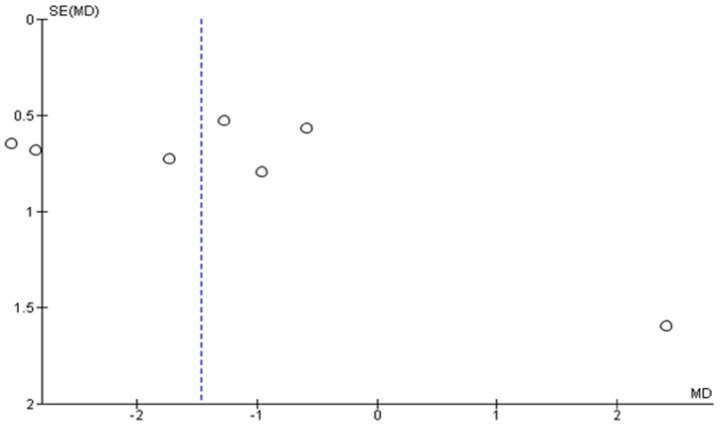
Funnel plot summary for primary outcome qualitative pain/burning sensation reduction assessment (VAS) from baseline up to the final follow-up timepoint.

**Figure 8:**
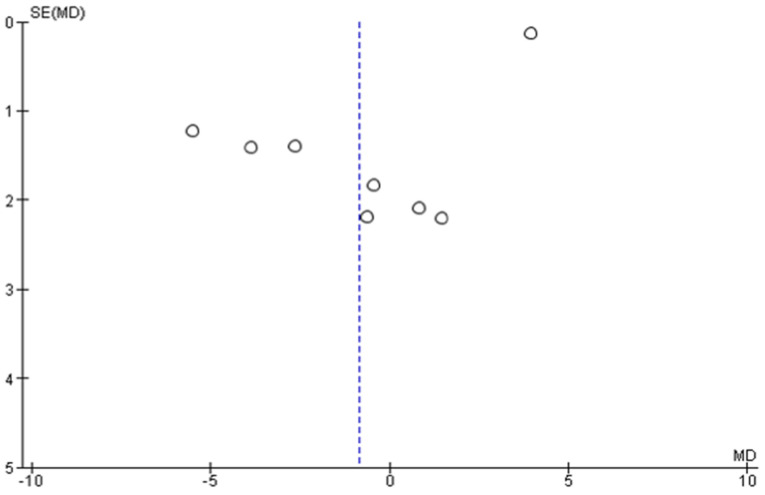
Funnel plot summary for secondary outcome qualitative anxiety/depression and QoL assessment (OHIP) from baseline up to the final follow-up timepoint.

A high heterogeneity and asymmetry in the funnel plots (Figure 7 and Figure 8) were noted in the meta-analytical assessment of the studies, which were eligible in this review. Hence, there is a significant risk of reporting bias in the results of this meta-analysis [89]. However, a subgroup or sensitivity analysis could not be performed owing to the low sample size and poor study quality resulting in a high RoB which was detected in the qualitative bias assessment of the eligible studies in the meta-analysis [75,79,82].

## 4. Discussion

The eligible RCTs of this systematic review and meta-analysis that enrolled subjects diagnosed with npBMS, according to various criteria [1,4,86,87] and treated with PBMT (laser-PBM or LEDs-PBM) of various wavelengths compared to placebo or pharmacotherapy were examined and dissected. Notwithstanding the discrepancies and variations in the published PBM parameters, study design, variable assessment tools and missing data, the greater number of the eligible studies have stated positive results, favouring PBMT effectiveness in BMS management. Therefore, our systematic review and meta-analysis, for the first time, has tackled methodological reproducibility and PBM protocols standardisation by offering suggested recommendations based on authors’ expert opinion and evidence-based science and practice, paving a strategic framework for purposes of future extensive PBM research in pBMS management. Within this perspective, our concise and comprehensive review has unveiled the fundamental RCTs’ shortfalls and drawbacks and provided scientific evidence-approach in science and practice to overcome them, which are listed below:

### 4.1. Role of RoB Assessment

Overall RoB assessment revealed that 33.3% of the included studies (four studies) [74,77,78,84] were at a low risk of bias, 8.3% (one study) [76] and 58.3% of the included studies (seven studies) [75,79,80,81,82,83,85] were at a high risk of bias, respectively. Majority of the high RoB derived from the randomisation process (4 out of 12 studies) [79,80,81,83], deviations from intended interventions (6 out of 12 studies) [79,80,81,82,83,85] and measurement of the outcome (6 out of 12 studies) [75,79,80,81,82,83]. Furthermore, none of the eligible studies indicated the presence of a potential conflict of interest.

### 4.2. Role of Meta-Analysis Outcome

The authors of the present systematic review and meta-analysis needed to explore the effectiveness or superiority (if any) of PBMT with LEDs or lasers compared to placebo/control (sham PBMT) in the management of outcome variables such as: pain, functional improvement, anxiety/depression, QoL and overall treatment satisfaction in patients with BMS. This led the authors to perform a critical appraisal of the available scientific evidence. After thoroughly scrutinising the available data, 12 studies qualified for this systematic review and meta-analysis [74,75,76,77,78,79,80,81,82,83,84,85].

Owing to the scarcity of the available numerical data and several methodological inconsistencies, only 5 out of 12 studies were eligible for a meta-analysis [74,75,79,82,84]. Only 4 out of 12 eligible studies had relevant numerical data and they contributed to the meta-analytic findings for the primary outcome measure (pain reduction assessment by qualitative measurement with VAS) [75,79,82,84]. The findings from the above four studies comprising a total of 236 participants were scrutinized from baseline up to the final follow-up evaluation for each study. The findings revealed a SSID favouring PBMT group compared to sham PBMT, although with substantial high inter-study heterogeneity [75,79,82,84].

The role of QoL, as vital indicator of how an individual’s overall well-being is affected by disease/disorder/disability in their daily life was assessed in 7 out of 12 studies [74,75,76,77,79,82,84]. With regards to the secondary outcome measure (anxiety/depression and QoL assessment by qualitative measurement with OHIP), relevant numerical data were available in 5 out of 12 studies [74,75,79,82,84]. The results obtained from the above five studies comprising a total of 321 participants were evaluated from baseline up to the final follow-up evaluation for each study. In coherence with the findings for the VAS assessment, the findings for anxiety/depression and QoL assessment also revealed a SSID favouring PBMT group compared to sham PBMT with substantial high inter-study heterogeneity [74,75,79,82,84]. A high heterogeneity and asymmetry in the results could be co-related with the funnel plot assessment. However, a subgroup or sensitivity analysis which would have helped to exclude the outlier studies, could not be performed owing to the low sample size of the eligible cohort and poor study quality resulting in a high RoB which was detected in the qualitative bias assessment of the majority eligible studies in the meta-analysis [75,79,82].

Sun and Jiang (2019) [54] conducted a systematic review and meta-analysis on 15 clinical trials (six randomised trials and nine RCTs) in order to assess the efficacy of PBMT with or without placebo for relieving pain/burning sensation in patients diagnosed with BMS. The authors have concluded that PBMT was effective in BMS and PBMT with 790 nm wavelength particularly the most efficacious. Since the laser power parameters varied widely in the included studies in this review the authors have failed to provide a clear conclusion while emphasising the need for further RCTs.

Zhang et al. (2020) [53] have performed a systematic review on 12 RCTs involving 547 patients in order to investigate the effect of PBMT on pBMS as compared to placebo. The outcome variables were pain reduction and QoL improvement. The authors performed a meta-analysis to assess pain reduction with PBM in five trials and found that PBM was effective in reducing pain compared with placebo. They also conducted a meta-analysis on seven groups in four trials and showed that in comparison to placebo, PBM was effective in improving QoL.

**Figure 9:**
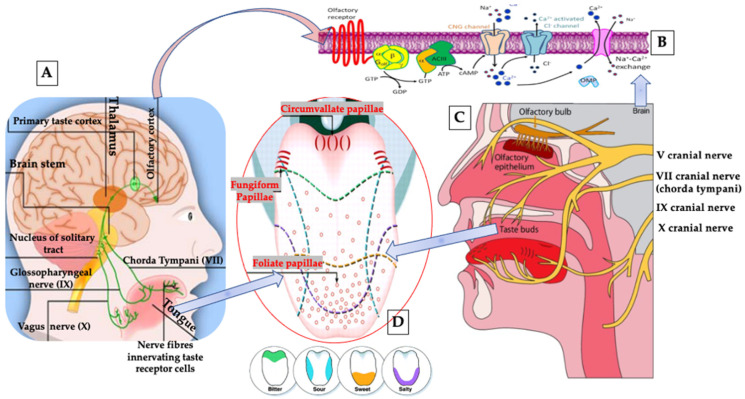
(**A**–**D**) Shows the correlation between taste and smell senses dysfunction in patients with burning mouth syndrome (BMS), even though taste and smell are separate senses with their own receptor organs, they are intimately entwined. Olfactory information passes to adjacent parts of the orbital cortex, where the combination of odour and taste information helps create the perception of flavour [100,102,103]. As shown in (**A**), taste signals go from the mouth, via cranial nerves, to the medulla oblongata in the brainstem, then up to the thalamus and on to the cortex, where the sensation becomes a perception. The distribution of trigeminal nerve (V3), glossopharyngeal nerve (IX), Vagus nerve (X) and chorda tympani (branch of the facial nerve (VII)) innervating the tongue. As shown in (**B**), shows the mechanism of action of smell sense where the olfactory bulb connects directly to the limbic system, the brain area that regulates emotion. As shown in (**C**), the distribution of the V3 nerve. As shown in (**D**), the distribution of the of four basic tastes (sweet, bitter, sour, salty) on the tongue according to their associated papillae (circumvallate, fungiform, foliate). Sweet, salty and bitter tastes had higher thresholds, but the sour taste had lower thresholds. Sour is the taste that involves the activity of H+ ions directly through channels in the receptor membranes, which also can activate small pain fibres. In addition to peripheral nerve degeneration, a more sensitive perception of acids (for taste and pain) could be related to the peripheral mechanism of BMS. List of the abbreviations are listed in Supplementary File S2.

**Figure 10:**
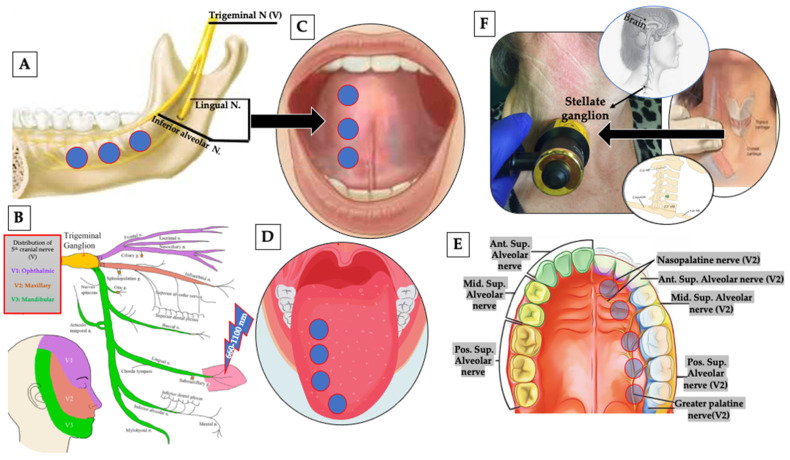
(**A**–**F**) Schematic representation of the proposed suggested number and allocations of the trigger points for PBM irradiation in unilateral BMS management. They are based on evidence derived from literature and expert opinion and are intended only to provide clinical guidance and serve as a starting point for extensive research. The blue circle represents the trigger points allocations and their number for unilateral symptoms. In case of bilateral symptoms, the same number of trigger points applies on both sides. As shown in (**A**), the allocation of the trigger points along the distribution of the lingual nerve (N), chorda tympani (branch of the facial N.) and inferior alveolar N, where the rationale number of the trigger points along the distribution of each nerve is three, depending on the diameter of the beam. As shown in (**B**), the trigeminal ganglion where V3 branches [ophthalmic (V1), Maxillary (V2), mandibular (V3)] emerge and their associated innervations. Irradiation of the ventral (**C**) dorsal (**D**) surfaces of tongue with wavelengths between 660–110 nm. As shown in (**E**), the hard and soft palate and their innervations as well the distribution of the trigger points along the distribution of the associated nerves. With regards the upper buccal mucosa of anterior, middle and posterior teeth (target areas), the allocation of the trigger points along the distribution of the nerves for their respected areas (**E**). As shown in (**F**), the extraoral approach of irradiation targets the stellate ganglion to reduce the abnormally increased sympathetic activities. Additionally, it illustrated in (**F**) the stellate ganglion landmark technique: The patient sits in a supine position with slight extension of the neck. The head is turned to the opposite side, applying the laser or LED probe on the meeting point of the clavicle with sternocleidomastoid muscle. List of the abbreviations are listed in Supplementary File S2.

Apart from there being an updated scientific appraisal on the role of PBMT in the management of patients diagnosed with BMS (search timeline between 1 January 2010–28 February 2021), the present systematic review and meta-analysis provides a critical and in-depth analysis of various vital parameters, which plays a role PBMT application. Moreover, the results of the present systematic review and meta-analysis of 12 studies confirm that PBMT is effective in the management of BMS and these results are in accordance with the results of the abovementioned pre-existing reviews [53,54]. However, it must be noted that the precision of the results obtained in this review are overshadowed by the poor methodological quality of majority of the eligible studies (seven studies) [75,79,80,81,82,83,85]. This finding along with a low sample size of studies with relevant numerical data eligible for meta-analysis, have greatly impaired the validity of the latter. Hence, in accordance with the conclusions of the existing systematic reviews and meta-analysis [53,54], the authors believe that research in the future should focus on conducting well-designed RCTs in order to determine the effectiveness of PBMT in patients diagnosed with BMS. In this context, a robust study design and methodology including a randomisation based on an unbiased assessment of outcomes is a necessity to ensure standardisation and reproducibility for future studies. Therefore, in the below sections, the authors have answered the focused review questions and have proposed suggested recommendations for clinical PBMT protocols for future extensive RCT studies for neurological primary BMS, which are justified on the current available evidence-based clinical practice and experts in the field (Table 5 and Table 6) (Figure 9 and Figure 10).

### 4.3. Methodology Quality

#### 4.3.1. Subjects Characteristics

It is interesting to note that the majority of the subjects who enrolled in our study were female and middle-aged. This has been well-reported in the literature. Four studies included more than 50% females [77,79,80,82], while one study each included equal numbers of male and female patients [74] and only female patients [76], respectively. Only one study reported the patients’ racial background as Caucasian [77], whereas all other studies in this cohort failed to report the relevant data [74,76,79,80,82]. It is important to highlight that the optical properties in terms of oral mucosa colour, phenotype, consistency and composition, as well the location, play a crucial role in PBM optimal outcome. This related to the absorption and scattering phenomena when the light travels through different layers can lead to loss part of the energy [90].

#### 4.3.2. Evaluation of Areas of BMS Presented Symptoms

There is a lack of fundamental clarity in the inclusion and exclusion criteria regarding patients’ symptoms, whether unilateral or bilateral among the majority of the included studies (Table 2). This plays a vital role when mapping the target area for trigger points allocation. Additionally, the number of recruited subjects were uneven with the study groups (Table 2). Moreover, it is important to differentiate between BMS symptoms area and other affected areas to ensure mapping the trigger points effectively (explained in Section 4.3.5.) (Figure 10).

#### 4.3.3. Diagnostic Criteria

Diagnosis of neurological primary BMS remains a challenge for health professionals due to the discrepancy between the intensity of pain as reported by the patient and the absence of objective clinical lesions [91]. Hence, standardised diagnostic criteria are essential to ensure optimisation of the treatment modality, and ultimately the clinical outcomes. Therefore, international classification of orofacial pain (ICOP) [92] has classified BMS as an idiopathic orofacial pain and described it as “an intraoral burning or dysaesthesia sensation, recurring daily for more than 2 hours per day for more than 3 months, without evident causative lesions on clinical examination and investigation”. The ICOP has further suggested the use of somatosensory assessment to subgroup BMS into ‘with or without somatosensory changes” [92].

Within our review eligibility criteria, we have not specified subjects diagnosed primary BMS diagnostic according to certain diagnostic criteria, aiming to maximise the number of the included RCT and to observe, whether specific diagnostic criteria would offer better results. We have analysed the results of the included studies based on their utilised BMS diagnostic criteria as follows: IASP, Revised 2013 [86], IASP, 2016 [87], ICHD-3, 2nd edition, 2013 [4] and ICHD-3-D, 3rd edition, 2018 [1], which defines primary BMS, according to the ICOP. Only 1 [84] out of 12 eligible studies utilised ICHD-3-D, 3rd edition, 2018. Due to the small number of included studies, we could not conclude whether the diagnostic criteria have influenced the clinical outcomes optimisation.

In chronic pain conditions, Opiorphin could serve as a universal objective indicator. In this context, opiorphin can reflect emotional and socio-relational imbalances occurring with BMS, hence can represent BMS biomarker. Further understanding of opiorphin’s involvement in pain pathways can add value in developing new BMS clinical diagnostic methods. The levels of Opiorphin can be measured by HPLC-MS/MS method (Table 5) [93]. Two recent consensus papers have published guidelines for BMS diagnostic criteria [94,95].

#### 4.3.4. Evaluation of Outcome Measures Assessment

Standardised, valid and robust outcome assessment tools play a vital role in outcomes optimisation. Our review scrutinised the results of the included studies and showed a high bias level and heterogeneity related to poor quality of methodology (Figure 5, Figure 6, Figure 7 and Figure 8). Hence, in this section, the authors propose suggested recommendations of outcomes assessment tools based on evidence-based practice, which can be utilised in future BMS extensive studies [74,75,76,77,78,79,80,81,82,83,84,85,93,96,97,98,99,100,101,102,103,104,105,106,107,108,109,110] (Table 5).

The following salivary biomarkers can be evaluated as diagnostic and objectively outcome assessment measures, as the innate immune system of patients with BMS can be altered [96]: complement C4 (CC4), α1-antitrypsin (a1AT), C-reactive protein (CRP), macrophage inflammatory protein-4 (MIP4), pigment epithelium-derived factor (PEDF), serum amyloid P (SAP), haptoglobin (Hp), a panel of biomarkers of oxidative stress integrated by uric acid and ferric reducing activity of plasma (FRAP), the salivary alpha-amylase (sAA) as a biomarker of the adrenergic system and total immunoglobulin A (IgA).

Pain and stress of the patients with pBMS was measured by VAS and HAD score and their influence of oral health [96] (Table 5).

The salivary flow rate can be objectively assessed by quantifying the unstimulated and stimulated whole saliva, using sialometry to evaluate the salivary secretion IgA (SIgA) [97] and unstimulated salivary flow rate [100]. Additionally, salivary TRPV1 and NGF levels and purinergic receptors P2X3, oxidative stress and antioxidants status are useful tools to evaluate objectively the effectiveness of PBMT [99] (Table 5). PBM effects in reducing the salivary levels of TNF-α and IL-6, which are proinflammatory mediators found to be elevated in patients with BMS. This was supported by an RCT conducted by Pezelj-Ribaric et al. (2013) [81]. Biological markers and proinflammatory cytokines such as IL-2 and IL-6 were increased in BMS which response to treatment.

Evidence supports the theory that the neuropathic mechanisms underlying BMS involve the somatosensory, gustative and olfactory pathways [100] (Figure 9). A systematic review and meta-analysis conducted by Pereira et al. (2021) [101] showed the influence of loss of the pleasure of eating caused by BMS which had a negative impact upon QoL. Altered taste (bitter) decreased sensitivity to sweet, salt, phantom taste and burning sensation [102]. A decrease in the gustatory sensitivities of the tongue tested by an electrogustometry on the dorsal tongue has proposed degeneration of chorda tympani nerve, resulting in trigeminal neuropathy or glossopharyngeal nerve inhibition [103] (Figure 1 and Figure 9). None of the included studies have evaluated the taste sense specifically, as well the smell.

The authors of this review suggest the use of the taste alteration scale developed by Kano T et al. (2013) for chemotherapy-induced taste alteration scale [104], which is composed of 18 items, evaluated on a five-point type scale, and divided into three dimensions: quantitative and quantitative changes in the perception of flavours and problems related to nutrition [105]. An 18-item scale was developed with four dimensions identified through factor analysis: decline in basic taste, discomfort, phantogeusia (metallic or salty taste) and parageusia (complete less of taste) and general taste alterations (Table 5).

It has been well-reported that patients with pBMS have distinct differences in somatosensory function (Table 5), implying a complex pathophysiology and interaction between nociceptive processing impairments and psychologic functioning [106]. It would be indicative to employ a quantitative sensory testing (QST) protocol-including the following, as diagnostic tests prior, conducting a study, in order to recruit a homogenous BMS cohort: cold pain threshold (CPT), cold detection threshold (CDT), thermal sensory limen (TSL), warmth detection threshold (WDT), heat pain threshold (HPT), paradoxical heat sensation (PHS), wind-up ratio (WUR), mechanical pain threshold (MPT) and pressure pain threshold (PPT).

The authors of this review suggest employment of Patient Reported Outcomes (PROMs) which are the tools and/or instruments that have been developed to ensure both a valid and reliable measurement of BMS-PROMs such as: QoL measures and health-related behaviours such as: anxiety and depression [107,108] (Table 5). Additionally, Initiative on Methods, Measurement and Pain Assessment in Clinical Trials (IMMPACT) was updated two years after [109] to IMMPACT-II which lists six domains for assessing patients with chronic pain (Table 5), which involves pain measurement, physical and emotional aspects, reports of adverse events, patients’ perception of the results of the treatment and adherence to it. Interestingly, magnetic resonance imaging (MRI) and arterial spin labelling prefusion MRI is a reliable tool to evaluate the outcome of BMS treatment by measuring the grey matter volume and cerebral blood flow [110].

**Table 5:** Illustrates the qualitative and quantitative measurements for primary and secondary outcomes utilised in the selected studies of this review and further suggested quantitative assessments such as: salivary analysis, immunohistochemistry and MRI [92,93,96,97,98,99,100,101,102,103,104,105,106,107,108,109,110]. Abbreviations: IMMEC: Initiative on Methods, Measurement and Pain Assessment in Clinical Trials; SSI: symptoms’ severity index; BDI: Beck depression inventory; CITAS: chemotherapy-induced taste alteration scale; QST: quantitative sensory testing; QualST: qualitative sensory testing; PSFS; patient specific functional scale; IgA: immunoglobulin A; CRP, C-reactive protein; a1AT: α1-Antitrypsin; PEDF, pigment epithelium-derived factor; SAP: serum amyloid P; MIP4: Macrophage Inflammatory Protein-4; CC4: complement C4; CB1: cannabinoid receptors type 1; LC-MS-MS: liquid chromatography with tandem mass spectrometry; NPRS: Numerical pain rating scale; MRI: magnetic resonance imaging. List of the abbreviations are listed in Supplementary File S2.

Assessment of Outcome Measures		Primary Outcomes	Secondary Outcomes		
		Pain Reduction	Functional Improvement	Anxiety/Depression and QoL	Over All Treatment Satisfaction
Patient-reported outcomes (PROMs/IMPACT)	Qualitative (Subjective)	VAS, NPRS, SSI IMMEC, PPI	PSFS 12-indicies: Functional Problems Questionnaire	BAI, PAD, HRQL, OHIP-14	
	Quantitative (Objective)	BPI, MPQ	Functional problems assessment	BDI, HADS, Euro Qol-5D 5L, GDS, SF-36, SCL-90-R	PGI-I
Trigeminal somatosensory assessment	Combined qualitative and quantitative		CITAS (taste), QST/QualST		
Immuno-histochemistry	Quantitative		Spectrophotometric method: IL-8, IL-1β, IL-6, IL-2, TNF-α TNF-α (pg/ml), NGF, TRPV1, CB1, oxidative stress markers. ELISA (UWS) Sialometry (UWS pH) LC-MS-MS: Opiorphin,		
Salivary analysis profile	Quantitative		Salivary flow rate: CC4, IgA, IgG, IgM, lysosomes, a1AT, CRP, MIP4, PEDF, SAP, Calcitonin level [Calcitonin gene-related peptide (CGRP) modulates nociceptive trigeminovascular transmission] Unstimulated salivary flow rates (SFRs)		
Microcirculation assessment	Quantitative		Videocapillaroscopy evaluating the capillary bed: parametric data (Capillary loop length, diameter, density and tortuosity) and non-parametric data (Presence of capillaries with particular morphology)		
MRI	Quantitative		Alterations in gray matter volume (GMV) using structural MRI and cerebral blood flow (CBF), using and arterial spin labeling (ASL) perfusion MRI		

#### 4.3.5. Assessment of the Number and Allocations of the Trigger Points of the Affected Areas

At the root of the tongue, the circumvallate papillae are distributed for which its taste buds receive bitter signals from glossopharyngeal nerve sensory fibres. Whereas, the foliate papillae are found on the lateral borders of posterior one-third of tongue where their tasted buds react primarily to sour taste, innervated by branches of chorda tympani and glossopharyngeal nerve, while the palatine taste buds are innervated by the facial nerve branches [111]. It is noteworthy that the affected areas are related to the nerves that innervate the target tissue and the trigger points should be mapped along the distribution of those nerves (Figure 10) to maximise optimisation of the clinical outcomes.

The concept of approaching the sympathetic system through the stellate ganglion block with epinephrine has been well-documented in reducing pain in patients with neurological pPBM [112]. A comparative study between PBMT and control/sham conducted by Nakase et al. (2004) [113] and showed that stellate ganglion irradiation (SGR) with 600–1600 nm, including a combination of red and NIR irradiation at power output of 1500 mW, 10 min exposure time, total energy density: 194.8 J/cm^2^, stellate ganglia trigger point (one point). They concluded that SGR is an effective treatment for glossodynia, as SGR inhibits abnormally increased sympathetic activity associated with glossodynia and stabilising the tongue blood flow, thereby alleviating pain. None of the included studies in this systematic review have utilised this extraoral approach, neither as a single or combined therapy with intraoral approach. The authors propose to consider extraoral Stellate ganglion irradiation combined with intraoral approach (Figure 10).

Based on the above note, the authors proposed suggested irradiated trigger points and affected areas to optimise the clinical outcomes (Figure 10).

### 4.4. Assessment of Reported PBM Parameters and Treatment Protocol

A persistent inconsistency in delivering valid, reliable PBM doses (fluence) to the target tissues was identified. The lack of consensus in delivering a standardised protocol for PBMT, is partly due to the lack of adequate reported data and unreliable methods of assessment, which are fundamental for individual studies replication and protocol reproducibility.

Despite the lack of standardised protocols of application in the studies analysed, as RCTs with higher quality and lower risk of bias, several coincidences are found. The authors can suggest the following PBM protocol, as a proposed guide for future extensive research: wavelength in range of NIR, a power between 200 mW and 4 W, a beam area of 0.28 cm^2^ in CW emission mode, energy of 6 J per point, 30–60 s/point and total of 10 sessions, which is based on two sessions per week for 5 consecutive weeks.

Wavelength is an important PBM parameter in determining the depth of laser irradiance penetration reaching the target tissue, taking into consideration the absorption and scattering coefficients, which are higher in shorter wavelengths. Additionally, developing evidence implies an increase in glutamate level in patients with neuropathic pain [114]. Red and NIR light can induce intracellular Ca^2+^ flux via activation of glutamate and N-methyl-D-aspartate receptors (NMDA) receptors and modulate the level of glutamate in NP model, resulting in an analgesic effect [115].

There is a dose-related response which is best described as a multiphasic outcome, as at relatively low doses of radiant exposure, there can be photobiostimulation associated with enhanced healing, whereas at higher levels, photobioinhibition can be associated with optimal pain relief [116,117]. In this context, many factors play a vital role in clinical outcome success including variations in the anatomy and site location of the target and the clinical condition. In order to achieve a predictable and an optimal outcome, an appreciation of these factors and further understanding of laser parameters, tissue optical properties and target-seated depth to deliver an adequate dose are essential to consider [118].

Many studies have shown the effectiveness of utilising a flattop beam profile to ensure equal distribution of the photonic energy over 1 cm^2^ of surface area of the target tissue versus gaussian beam profile [43,90,119]. This could be an approach to be utilised for future studies.

Table 3 illustrates the percentage (%) of missing data related to PBM parameters and protocols in the included studies, which cements the heterogeneity of the data and lack of reproducibility. Despite only three studies utilising a power meter, they showed a high risk of bias and heterogeneity. This could be related to a poor quality of methodology and short-term follow-up timepoints. The authors suggest that future studies need to adhere reporting the essential and desirable laser treatment parameters, as well power meter utilisation to ensure standardised and reproducible protocols for future studies, which are presented in Table 6 [120].

**Table 6:** Represents the essential and desirable laser treatments that should be reported to standardise PBM protocol and improve methodology reproducibility among clinicians and facilitate the comparison of results among researchers. Adapted with permission from ref. [120], Copyright 2011 Mary Ann Liebert. List of the abbreviations are listed in Supplementary File S2.

Device Information	Essential Reported Parameters		Desirable Reported Parameters
	Irradiation Parameters	Treatment Parameters	Energy per Pulse (J)
Manufacturer	Wavelength (nm)	Beam spot size at target (cm^2^)	Polarisation
Model identifier	Spectral bandwidth (nm)	Irradiance at target (mW/cm^2^)	Aperture diameter (cm)
Emitters Type (e.g., nGaAlP LED, GaAlAs LASER, KTP LASER)	Operating mode (CW, pulsed, super pulsed)	Exposure duration (sec)	Irradiance at aperture (mW/cm^2^)
Number of emitters	Frequency (Hz)	Radiant exposure (J/cm^2^)	Beam diverange (°)
Spatial distribution of emitters. (e.g., 4 emitters spaced 2 cm apart in a square pattern).	Pulse width (second)	Radiant energy (J)	Beam shape
Beam delivery system (e.g., fibreoptic, free air/scanned, hand-held probe).	Duty cycle (%)	Number of points irradiated	Scanning technique
	Beam profile	Area irradiated (cm^2^)	Speed of movement
		Application technique	
		Number and frequency of treatment sessions & total radiant energy (J)	

## 5. Conclusions

Up to date, this is the first extensive systematic review of 12 studies and meta-analysis of four studies that synthesised an eclectic assortment of experimental protocols. Despite the positive results of the included studies in this review favouring PBMT in neurological primary BMS, high RoB and heterogeneity due to a small sample and poor quality of methodology were noted. This review highlighted the drawbacks and gaps of the included studies results. Hence, for the first time, we have suggested recommendations for both clinical PBMT protocols and reproducible methodology, which are ultimately the first stepping-stone for evidence-based consensus. Additionally, standardised diagnostic criteria for neurological primary BMS are required for future studies, as well understanding the genetic part of BMS to facilitate optimisation of PBMT.

## Figures and Tables

**Table 1:** Illustrates the qualitative and quantitative measurements for primary and secondary outcomes utilized in the selected studies of this review. Abbreviations: VAS: visual analogue scale; NSP: Numerical scale of pain; PPI: present pain intensity; PAD: pleasure–arousal–dominance; BAI: beck anxiety inventory; HRQL: health-related quality of life; MPQ: McGill Pain Questionnaire; QHIP-14: health impact profile-14; HADS: Hospital Anxiety And Depression Scale; PGI-I: patient global impression of improvement; GDS: geriatric depression scale; SF-36: short Form-36 Health Survey Questionnaire; SCL 90-R: Symptom Checklist-90-R; UWS pH: unstimulated whole salivary flow-pH; ELISA: enzyme-linked immunosorbent assay. List of the abbreviations are listed in Supplementary File S2.

Assessment of Outcome Measures		Primary Outcomes	Secondary Outcomes		
		Pain/Burning Sensation Reduction	Functional Improvement	Anxiety/Depression and QoL	Over All Treatment Satisfaction
Patient-reported outcomes	Qualitative (Subjective)	VAS, NSP, PPI		BAI, PAD, HRQL, OHIP-14 (all versions)	
	Quantitative (Objective)	MPQ		HADS, SCL-90-R, Euro Qol-5D 5L, GDS, SF-36	PGI-I
Salivary analysis profile	Quantitative		Sialometry (UWS pH), TNF-α and IL-6, ELISA (Unstimulated saliva)		
Microcirculation assessment	Quantitative		Videocapillaroscopy evaluating the capillary bed: parametric data (capillary loop length, diameter, density and tortuosity) and non-parametric data (Presence of capillaries with particular morphology)		
Immuno-histochemistry analysis	Quantitative		Il-8, IL-1β, IL-6, IL-2, TNF-α		

**Table 2:** Tabular description of all the selected eligible of vivo RCTs human studies, summarising the demography, study design, symptoms, diagnostic criteria, functional problems, affected areas, intervention groups, methods of assessment, evaluation period and outcomes. Abbreviation: RCTs: randomized controlled trials, SB: Single-blind; DB: double-blind; ADH: anxiety and depression scale, OHIP-14: oral health impact profile; VNS: visual numeric scale; VAS: visual analogue scale; PPI: present pain intensity scale; NRS: numerical rating scale; HADS, hospital anxiety–depression scale; PGI-I, patient global impression of improvement; ELISA: enzyme-linked immunosorbent assay; GDS: geriatric depression scale; BMS: burning mouth syndrome; SOB: secondary oral burning; TNF-α: transforming necrotic factor-alpha; L: lip; TT: tip tongue; LT: lateral tongue; DT: dorsal tongue; BM: buccal mucosa; LM: labial mucosa; HP: hard palate; SP: soft palate; G: gingivae; AM: alveolar mucosa; VM: vestibular mucosa; SM: sublingual mucosa; UL: upper lip; LL: lower lip; LG: lower gingivae;: MR: mandibular ridge; QoL: quality of life; 1/12: 1 month; 2/12: 2 months; 3/12: 3 months; 4/12: 4 months; 6/12: 6 months; QoL-OH: QoL related to oral health; vs: versus; OHIP-CRO 14: Croatia version of OHIP-14; 3/52: three weeks; 8/52: 8 weeks; 12/52: twelfth weeks: 12/52; COB: capillary oral bed; 14/7: 14 days; μ: micron; yrs: years; UWS: unstimulated whole salivary flow; SD: standard deviation; MPQ: McGill Pain Questionnaire; vs: versus; SF-36: Short Form 36 Health Survey Questionnaire; SCL 90-R: Symptom Checklist-90-R; COB; infrared: IR; near-infrared: NIR. List of the abbreviations are listed in Supplementary File S2.

Study, Year, Origin and Citation	Journal Name/ Impact Factor (IF)	Study Design	Presented Symptoms and Duration	Diagnosis	Affected Area (s)	Functionality Problem (s)	Sample Size (*n*)	Gender Male (M), Female (F)	Age (yrs) (Mean ± SD)	Intervention Groups and Subject, No. Allocation	Evaluation Period	Assessed Parameter (s)	Evaluation Methods	Outcome and Conclusion
Bardellini et al., 2019 (Italy) [74]	Med Oral Patol Oral Cir Bucal (IF: 1.71)	RCT/DB	Pain Burning sensation, 6/12	IASP- 2016	TT, LT, DT, UL, LL, BM	Functional limitation, physical pain, psychological and social disabilities	85 (F)	F: 42 (G1) F: 43 (G2)	G1: 59 ± 9.51 G2: 60.86 ± 10.02	G1 (LLLT): 43 G2 (Placebo): 42	At baseline, mid-treatment (5th Session), End-treatment, 1/12 after treatment	Pain, Functionality limitation, Stress/ anxiety, Physical activity,	VAS Italian- OHIP	On VAS: in G1, a significant reduction in pain (*p* = 0.0008) and improvement in QoL-OH (*p* = 0.0002). VAS: at 5th session, a reduction in pain but no statistically significant differences between G1 and G2 (*p* = 0.6232). At end-treatment: statistically significant reduction in symptoms in G1 (*p* = 0.0008) and kept at 1/12 follow-up (*p* = 0.0005). On OHIP: G1 9.00 ± 4.20 vs. G2 −4.87 ± 3.75
Valenzuela et al., 2017 (Spain) [75]	J Oral Rehabil (IF: 2.4)	RCT/ Prospective/partially blinded/single centre	Oral burning/Pain, ≥6/12	IHS-2013	NI	Pain, Oral burning sensation. Reduction in saliva flow	44	M: 3 F: 41	65.5	G1 (LLLT): 16 (4 J) G2 (LLLT): 16 (6 J) G3 placebo/ sham laser: 12	At baseline, 2/52 and 4/52	Pain, oral health, salivary flow, anxiety/ depression, over all treatment satisfaction	VAS, OHIP-14 (Spanish version), Sialometry HADS, PGI-I	VAS and OHIP-14 scores reduced significantly over time of treatment in all groups. At 2/52 and 4/52: VAS and OHIP-14 for G1 and G2 significantly lower than in G3. No significant differences between G1 and G2. Xerostomia severity and HAD: no significant differences between groups. PGI-I: no significant differences G1 and G2. Overall VAS scores improvement from baseline to end-LLLT were: G1: 15.7%, G2: 15.6%, G3: 7.3%
Arbabi-Kalati et al., 2015 [76]	J Clin Exp Dent. (IF: 1.73)	RCT/SB	Pain, burning sensation, 4/12	IASP- 2016	10 areas of oral mucosa BM, T, FM, SP, HP	Taste disturbance Pain intensity	20	M: 0 F: 20	G1: 47.2 G2: 46.6	G1 (LLLT): 10 G2 (Placebo Sham): 10	At baseline and after treatment Follow-up: NI	Pain, QoL	VAS, Persian- OHIP	Statistically significant improvement in burning sensation in G1 *(p =* 0.004), compared to G2. QoL: statistically significant in G1 (*p* = 0.01). VAS: G1: −4.4 ± 3.0 G2: −0.2 ± 1.5. OHIP: G1: −15.0 ± 11.4 vs. G2: 0.3 ± 11.5
Arduino et al., 2016 (Italy) [77]	Lasers Med. Sci. (IF: 1.94)	RCT/comparative Double or single blind: NI,	Pain, burning sensation 6/12	IASP- 2016	Oral mucosa	Functional limitation, physical pain, psychological and social disabilities	33 Caucasian	M: 8 F: 25	G1: 68.5 G2: 65.4	G1 (LLLT): 18 G2 [Clonazepam (2 mg) lozenge]: 15	At 3/52, 8/52 and 12/52	Pain, QoL, PH saliva, anxiety/ depression	VAS, MPQ, PPI, OHIP-14, HADS, GDS, UWS pH	G1 was superior to G2, in improving pain intensity in all parameters but statistically significant only at 8/52 (*p* = 0.026) VAS: G1: −2.78 ± 4.08 vs. G2: −1.15 ± 1.80. MPQ: G1: −10.05 ± 4.80 vs. G2: −11.00 ± 4.80. OHIP: G1: −11.06 ± 32.10 vs. G2: 4.40 ± 43.00. No adverse effects in G1 but 32% of dizziness, fever, headache and lack apatite in G2
Sugaya et al., 2016 (Brazil) [78]	Braz Oral Res. (IF: 1.6)	RCT/SB	Burning sensation, dysgeusia, Duration: NI	IASP- 2013	Tongue, UL, LL, BM, MR SP; HP, LG	Xerostomia & dysgeusia	30 allocated Analysed only 23	M: 2 F: 21 (7 lost to follow-up)	59.7 (29–87)	Allocated: LG (LLLT): 15 CG (Placebo): 15, but analysed: LG: 13 CG: 10	At baseline, 15 mins after irradiation, At 14/7 1/12, 2/12 and 3/12	Pain	VAS, global perception chart pain index	A significant improvement of symptoms in LG over CG in two measurements only. Positive effect in emotional profile in LG and CG. CR: LG 6/13 vs. CG 4/10
Spanemberg et al., 2015 (Spain) [79]	J Biomed Opt. (IF: 3.17)	RCT/DB	Burning sensation, Pain, 6/12	IASP- 2016	TT, DT, LT (bilateral), UL, LL, HP, SP	NI	78	M: 11 F: 67	G1: 63.6 ± 9.61 G2: 60.5 ± 6.42 G3: 63.2 ± 6.91 G4: 61.5 ± 8.76	Three groups of LLLT vs. placebo G1: [LLLT IR (IR1W) ]: 20 G2: [LLLT-IR (IR3W)]: 20 G3: (LLLT red: 19. G4 (Placebo, Sham): 19	Baseline, end-treatment & 8/52 after treatment	Pain, QoL	VAS, VNS, OHIP-14	Significant improvement in symptoms and QoL (*p* < 0.01) in G1 compared to G4. On VS and VNS: G1 and G2 differed significantly compared to G4, but no significant difference between G3 and G4. OHIP-14: significant difference between G2 and G4. G1 and G3 didn’t differ significantly to G4
Skrinjar et al., 2020 (Croatia) [80]	Act Stomatol Croat. (IF: 0.75)	RCT/DB	Burning sensation, ≥3 months	IASP- 2016	Both sites: tongue, lip or HP	Xerostomia, intraoral disability	23	M: 3 F: 20	LLLT: 61 Placebo: 62	G1 (LLLT): 12 G2 (Placebo): 11	At baseline, end-treatment	Burning sensation, Salivary cortisol level	VAS, Unstimulated saliva (ELISA)	VAS scores and salivary cortisol levels were significantly lower in G1 and G2. LLLT was not better than placebo. No adverse effects reported for both groups
Pezelj-Ribarić et al., 2013 (Croatia) [81]	Lasers Med Sci. (IF: 1.94)	RCT/ unspecified	Burning sensation, pain Duration un-specified	IASP-2013	NI	NI	40	M: 13 F: 27	G1: 60.2 G2: 61.1	G1 (LLLT): 12 G2 (Placebo): 9	NI	Pain, UWS	VAS, TNF-α & IL-6 levels	On VAS: no significant differences in pain reduction between G1 and G2. In G1: no reduction of symptoms. VAS scores: G1: −4.2 vs. G2: −3. Decrease in TNF-α and IL-6
Sikora et al., 2018 (Croatia) [82]	Acta Clin Croat. (IF: 0.53)	RCT/SB	Burning mouth symptoms duration: NI	IASP-2016	NI	NI	44	M: 1 F: 43	Range 56–83 Mean age: 67.56	LLLT and Placebo but no data available	At baseline and after each treatment session	Pain, QoL	VAS, OHIP-CRO- 14	OHIP-CRO14: No significant differences between the groups prior and after LLLT (*p* > 0.05%). Neither of therapy protocols improved QoL scores. VAS score: significant decreases in both groups *(p <* 0.05%) and *(p <* 0.01%).
Spanemberg et al., 2019 (Spain) [83]	J Oral Medicine and Pathology (IF: 2.5)	RCT/DB	Pain/ burning, anxiety/ depression, 3/12	ICHD -2013	TT, LT, DT, BM, LM, HP, SP; G, AM	Intraoral & psychological disabilities	21	M: 1 F: 20	LG: 66.3 ± 7.52 CG: 66.4 ± 6.31	LG: 12 CG (sham): 9	At baseline, 8th session, 2/12 after treatment	Pain/ burning, dry mouth, dysphagia	VAS, HANDS	Initial VAS score mean was 8.9 in LG and 8.3 in CG (*p* > 0.05%). At end-treatment, VAS score was 5.5 in LG and 5.8 in CG. At 2/12, VAS score was 4.7 in LG and 5.1 in CG. Marginal significant improvement in dry mouth and dysphagia (*p* = 0.0538)
De Pedro et al., 2020 (Spain) [84]	Oral Diseases (IF: 2.6)	RCT/SB	Pain/burning, depression/ anxiety, lack of sleep >3/12	ICHD-3-2018	VM, L, BM, HP, LT, DT, SM	Intraoral disability, mental & psychological disabilities, lack of sleep	20	M: 2 for each group F: 8 for each group	LG: 66.30 ± 15.19 CG = 67.60 ± 10.68	LG: 10 CG (sham): 10	At baseline, 10th session 1/12 and 4/12 after treatment	Pain, sleepiness QoL, anxiety/ depression	VAS, SF-36, Psychometric SCL 90-R, MPQ, OHIP-14	On VAS: LG showed an improvement in pain at end-treatment and increased at 1/12 follow-up and continued to improve at 4/12 in (90%) (*p* = 0.013). In GC, 20% improvement at end-treatment and worsened in 40% at 1/12 and kept worsening in 40% at 4/12 follow-up. On McGill and OHIP-14: scores decreased in LG at end-treatment and maintaiend over the follow-up period, indicating a positive impact on psychological state. On mental health score: significant decrease in anxiety in LG at end treatment and at 4/12 follow-up. Statistical significant improvement in SF-36 scores in LG at 1/12 follow-up.
Scardina et al., 2020 (Italy) [85]	Dent J (Basel) (IF: NI)	RCT/DB	IO pain and burning sensation, >3/12	Unspecified criteria but specified burning sensation without specifying symptoms’ duration	ULM, DT, BM, LLM	Pain	40	Only F	62.06 ± 3.1	G1: LLLT: 20 G2: Placebo: 20	At baseline, after each treatment session (8 sessions) and 60 days after treatment	Pain, Capillary bed: Length, diameter, density, morphology tortuosity	VAS, NRS, Video-capillaroscopy evaluation	G1: a lasting improvement in symptoms. No statistical significant difference in COB in G2 (*p* > 0.05). Reduction in diameter of the following areas in G1: BM: 3μ, LL: 3μ, DT: 2μ. An increase in capillary length in all irradiated areas (*p* < 0.05). PBMT induced reduction in capillary diameter (long time period), reflected an improvement in clinical profile.

**Table 3:** Tabular representation of lasers/LEDs parameters utilised in the chosen eligible in vivo RCTs human studies related to burning mouth syndrome (BMS). Abbreviations: CW: continuous emission mode; TT: tip tongue; LT: lateral tongue; DT: dorsal tongue; BM: buccal mucosa; LAM: labial mucosa; HP: hard palate; SP: soft palate; G: gingivae; AM: alveolar mucosa; VM: vestibular mucosa; LM: lip mucosa; S: sublingual; EDT: entire dorsal tongue; EVT: entire ventral tongue; ARM: alveolar ridge mucosa; AT: apex tongue; T: tongue; LL: lower lip; UL: upper lip; MR: mandibular ridge; P: palate; LG: lower gingivae; FM: floor mouth; LLM: lower labial mucosa:; NI: no information; N/A: not applicable; mW: milliwatt; J: joule; μ: micron; cm^2^: square centimetre; 10/52: ten weeks; 2/52: 2 weeks; 5/52: 5 weeks; 4/52: 4 weeks; 9/52: 9 weeks; 10/52: 10 weeks; 2/52: 2 weeks; 14/52: 14 weeks; NI: no information; nm: nanometre; N/A: not applicable; Y: yes; min: minute; second: s; IO: intraoral; EO: extraoral; No.: number; mm: millimetre; ms: millisecond; W: week; IR: infrared; EO: extraoral; IO: intraoral. List of the abbreviations are listed in Supplementary File S2.

Study, Year, Origin And Citation	Light Source: Laser/LED (Symptoms’ Duration)	Emission Mode CW/ Gated/ Pulsed	Energy (J /Point )	Power Output (W/Mw)	Frequency & Pulse Width (PW)	Power Meter	Route of Irradiation (EO/IO) & no. of Trigger Points (TP)	Scanning Technique/Beam Profile	Contact (C)/ Non-Contact (NC)	Tip-Tissue Distance	Spot Size/ Fibre Tip Diameter	Fluence (Dose) (J/cm^2^)	Power Density (W/cm^2^)	Exposure Time/ Point Min/s	Frequency, Time Interval Between Sessions	Treatment Duration
Bardellini et al., 2019 [74]	660–970 nm (NI)	Pulsed/50%	NI	3200 mW	1–20,000 Hz PW:NI	NI	IO, TP(NI)	NI	NI	NI	1 cm^2^	NI	NI	3 mins and 51 s	Once a week	10/52
Valenzuela et al., 2017 [75]	GaAIAs laser 815 nm (NI)	CW	G1: 4J/point G2: 6J/point	1 W	N/A	NI	IO TP: 10	NI	C	NI	0.03 cm^2^	G1: 133.3 G2: 200	NI	G1: 4 s/point. G2: 6 s/ point	G1: Once a week G2: six times a week	G1: 4/52 G2: 4/52
Arbabi-Kalati et al., 2015 [76]	Diode laser 630 nm (NI)	NI	1 J	30 mW	N/A	NI	Total 10 TP (TP/site): T:2, FM:2, SP:1 and HP:1	NI	NI	NI	NI	1J/cm^2^/ area	NI	10 s	Twice a week	4/52
Arduino et al., 2016 [77]	Diode laser, 980 nm (NI)	CW	NI	300 mW	N/A	NI	NI	Spot/Gaussian	NC	2 mm	0.28 cm^2^ probe diameter: 0.6 cm	10	1	10 s/point	Twice a week (total 10 sessions)	5/52
Sugaya et al., 2016 [78]	IR-diode laser 790 nm (31.7 months)	CW	6 J/point	120 mW (0.12 W)	N/A	NI	24 sites for Laser G (T, LL, UL, BM, MR, P; LG). 17 sites for CG (T, LL, UL, BM, MR, P, LG)	NI	C	NI	0.03 cm^2^	6	4	50 s/point	Twice a week	2/52
Spanemberg et al., 2015 [79]	G1 and2: IR-laser 830 nm, G3: Red-laser 635 nm (6 months)	CW	G1 and G2: 5 J/point G3: 2 J/point	G1 and G2: 100 mW G3: 35 mW	N/A	Y	IO: AT: 3, LT: 4 DT: 10, BM: 8, LAM: 5, HP: 8, SP: 3, G and ARM: 3 each	NI	NI	NI	NI	G1 and G2: 176 G3: 72	G1 and G2: 3.57 G3: 1.25	G1 and G2: 50 s G3: 58 s	G1: 1 session/ week; G2: 3 session/ week; G3: 3 sessions/ week; CG: 3 sessions/ week	G1:10/52 G2: 9/52 G3: 9/52 G4: 9/52
Skrinjar et al., 2018 [80]	Ga-Al-As LED 685 nm (NI)	pulsed	NI	30 mW	5.20 Hz PW: NI	NI	3 reported burning sites (NI on number and location)	NI	NC	0.5 cm	3 cm^2^	2 (Total 60)	0.003	381 s/point	Daily for 10 days excluding weekend	10/7
Pezelj-Ribarić et al., 2013 [81]	685 nm	CW	NI	30 mW	NI	Y	Tongue mucosa, Number and allocation of TP: NI	NI	C	NI	2 mm, 1 cm^2^ surface area	3	NI	100 s/point	NI	NI
Sikora et al., 2018 [82]	GaAlAs laser 830 nm (NI)	Gated: 800 ms on/1 ms off, 80% duty cycle	NI	100 mW (average)	N/A	NI	NI	Slow circular movement/Gaussian	NC	5 mm	1 cm^2^	12	NI	5 mins/ session	Once per day (excluding weekend) (10 sessions)	14/7
Spanemberg et al., 2019 [83]	GaAIAs IR: laser 808 nm ± 5 nm (NI)	CW	3 J/point	200 mW	N/A	Y	Total: 41 (Bilateral) TP per site: TT: 3, LT: 4, DT: 10, BM: 8, LAM: 5, HP: 8, SP: 3, G or AM: 3	NI	NI	NI	0.088 cm^2^	NI	1.97	15 s /point	Twice a week (total eight sessions)	4/52
de Pedro et al., 2020 [84]	Diode laser 810 nm (NI)	CW	6 J/point	0.6 W	N/A	NI	IO: 56 points VM: 3 (4 sites), LM: 4, bilateral BM: 6/site, HP: 6, bilateral LT: 4/site, DT: 6, S: 4 bilateral	NI	NC	2 mm	0.5 cm^2^/300μ	12	1.2	10 s/ point	Twice a week (10 sessions in total)	5/52
Scardina et al., 2020 [85]	Diode LED 805 nm (NI)	NI	1200 J (total)	Total: 4 W	NI	NI	IO points: 4 areas BM, LAM, DT, LLM No. of TP unspecified)	Scanning/Gaussian	NI	4 cm Spacer used	NI	50	166.7 mW/cm^2^	300 s /area	Twice a week (eight sessions in total)	4/52
Missing data (%)	0% (90.90%)	27.27%	45.45%	0%	27.27%	81.81%	36.36%	81.81%	54.5%	72.72%	36.36%	27.27%	45.45%	18.18%	18.18%	18.18%

**Table 4:** Tabular description of all the selected eligible in vivo RCTs human studies of BMS, in terms of level of significance in subjective and objective assessments of pain, functionality improvement, anxiety reduction/QoL improvement and overall treatment satisfaction. Abbreviations: SS: Statistically significant; NSS: Not statistically significant; NI: No information. List of the abbreviations are listed in Supplementary File S2.

Study, Year, Origin and Citation	Primary Outcomes		Secondary Outcomes			
	Pain/Burning Sensation Reduction		Functional Improvement	Anxiety/Depression and QoL		Overall Treatment Satisfaction
	Qualitative (Subjective) VAS, NSP, PPI (SS, NSS, NI)	Quantitative (Objective) MPQ (SS, NSS, NI)	Quantitative (Objective) Salivary analysis Profile Microcirculation Assessment Immuno-Histochemistry Analysis (SS, NSS, NI)	Qualitative (Subjective) BAI, PAD, HRQL, OHIP (SS, NSS, NI)	Quantitative (Objective) HADS, SCL-90-R, Euro Qol-5D 5L, GDS, SF-36 (SS, NSS, NI)	Quantitative (Objective) PGI-I (SS, NSS, NI)
Bardellini et al., 2019 (Italy) [74]	SS	NI	NI	SS	NI	NI
Valenzuela et al., 2017 (Spain) [75]	SS	NI	NSS	SS	NSS	NSS
Arbabi-Kalati et al., 2015 [76]	SS	NI	NI	SS	NI	NI
Arduino et al., 2016 (Italy) [77]	SS	SS	SS	NI	SS	NI
Sugaya et al., 2016 (Brazil) [78]	SS	NI	NI	NI	NI	SS
Spanemberg et al., 2015 (Spain) [79]	SS	NI	NI	SS	NI	NI
Skrinjar et al., 2020 (Croatia) [80]	NSS	NI	NSS	NI	NI	NI
Pezelj-Ribarić et al., 2013 (Croatia) [81]	NSS	NI	NSS	NI	NI	NI
Sikora et al., 2018 (Croatia) [82]	NSS	NI	NI	NSS	NI	NI
Spanemberg et al., 2019 (Spain) [83]	SS	NI	NI	NI	SS	NI
De Pedro et al., 2020 (Spain) [84]	SS	SS	NI	SS	SS	NI
Scardina et al., 2020 (Italy) [85]	NSS	NI	NSS	NI	NI	NI

## Data Availability

Not applicable.

## Supplementary Materials

The following are available online at https://www.mdpi.com/article/10.3390/pharmaceutics13111838/s1, Supplementary File S1: PRISMA checklist; Supplementary File S2: List.
