# Supplementary material for: Role of Photobiomodulation Therapy in Neurological Primary Burning Mouth Syndrome. A Systematic Review and Meta-Analysis of Human Randomised Controlled Clinical Trials
Source: Pharmaceutics. 2021 Nov 2;13(11):1838. doi: 10.3390/pharmaceutics13111838 (PMC8624276; doi:10.3390/pharmaceutics13111838)
Supplement: Supplementary file 1 [file pharmaceutics-13-01838-s001.zip › pharmaceutics-1397693-supplementary.pdf]

pharmaceutics

Supplementary Materials: Role of Photobiomodulation Therapy in Neurological Primary Burning Mouth Syndrome. A Systematic Review and Meta-Analysis of Human Randomised Controlled Clinical Trials

Reem Hanna, Snehal Dalvi, Rene Jean Bensadoun, Judith E. Raber-Durlacher, and Stefano Benedicenti

Table S1. PRISMA checklist.

Section/topic	#	Checklist item	Reported on page #
**TITLE**			
Title	1	Identify the report as a systematic review, meta-analysis, or both.	1
**ABSTRACT**			
Structured summary	2	Provide a structured summary including, as applicable: background; objectives; data sources; study eligibility criteria, participants, and interventions; study appraisal and synthesis methods; results; limitations; conclusions and implications of key findings; systematic review registration number.	1
**INTRODUCTION**			
Rationale	3	Describe the rationale for the review in the context of what is already known.	4
Objectives	4	Provide an explicit statement of questions being addressed with reference to participants, interventions, comparisons, outcomes, and study design (PICOS).	4
**METHODS**			
Protocol and registration	5	Indicate if a review protocol exists, if and where it can be accessed (e.g., Web address), and, if available, provide registration information including registration number.	4
Eligibility criteria	6	Specify study characteristics (e.g., PICOS, length of follow-up) and report characteristics (e.g., years considered, language, publication status) used as criteria for eligibility, giving rationale.	4-6
Information sources	7	Describe all information sources (e.g., databases with dates of coverage, contact with study authors to identify additional studies) in the search and date last searched.	5,8
Search	8	Present full electronic search strategy for at least one database, including any limits used, such that it could be repeated.	7

Study selection	9	State the process for selecting studies (i.e., screening, eligibility, included in systematic review, and, if applicable, included in the meta-analysis).	6
Data collection process	10	Describe method of data extraction from reports (e.g., piloted forms, independently, in duplicate) and any processes for obtaining and confirming data from investigators.	5-8
Data items	11	List and define all variables for which data were sought (e.g., PICOS, funding sources) and any assumptions and simplifications made.	4,5,7
Risk of bias in individual studies	12	Describe methods used for assessing risk of bias of individual studies (including specification of whether this was done at the study or outcome level), and how this information is to be used in any data synthesis.	8
Summary measures	13	State the principal summary measures (e.g., risk ratio, difference in means).	7,8
Synthesis of results	14	Describe the methods of handling data and combining results of studies, if done, including measures of consistency (e.g., I^2) for each meta-analysis.	8

**Section/topic**	**#**	**Checklist item**	**Reported on page #**
Risk of bias across studies	15	Specify any assessment of risk of bias that may affect the cumulative evidence (e.g., publication bias, selective reporting within studies).	8
Additional analyses	16	Describe methods of additional analyses (e.g., sensitivity or subgroup analyses, meta-regression), if done, indicating which were pre-specified.	N/A
**RESULTS**			
Study selection	17	Give numbers of studies screened, assessed for eligibility, and included in the review, with reasons for exclusions at each stage, ideally with a flow diagram.	8

Study characteristics	18	For each study, present characteristics for which data were extracted (e.g., study size, PICOS, follow-up period) and provide the citations.	8
Risk of bias within studies	19	Present data on risk of bias of each study and, if available, any outcome level assessment (see item 12).	15
Results of individual studies	20	For all outcomes considered (benefits or harms), present, for each study: (a) simple summary data for each intervention group (b) effect estimates and confidence intervals, ideally with a forest plot.	8-27
Synthesis of results	21	Present results of each meta-analysis done, including confidence intervals and measures of consistency.	N/A
Risk of bias across studies	22	Present results of any assessment of risk of bias across studies (see Item 15).	20-27
Additional analysis	23	Give results of additional analyses, if done (e.g., sensitivity or subgroup analyses, meta-regression [see Item 16]).	24
**DISCUSSION**			
Summary of evidence	24	Summarize the main findings including the strength of evidence for each main outcome; consider their relevance to key groups (e.g., healthcare providers, users, and policy makers).	28-37
Limitations	25	Discuss limitations at study and outcome level (e.g., risk of bias), and at review-level (e.g., incomplete retrieval of identified research, reporting bias).	28-37
Conclusions	26	Provide a general interpretation of the results in the context of other evidence, and implications for future research.	37
**FUNDING**			
Funding	27	Describe sources of funding for the systematic review and other support (e.g., supply of data); role of funders for the systematic review.	N/A

Table S2. List of abbreviations (In alphabetical order).

Sr. no.	Abbreviation	Full form
1.	1/12	One-month
2.	2/12	Two-months
3.	3/12	Three-months
4.	4/12	Four-months;
5.	6/12	Six-months
6.	3/52	Three-weeks
7.	8/52	Eight-weeks
8.	12/52	Twelfth-weeks
9.	14/7	Fourteen days
10.	a1AT	α1-Antitrypsin
11.	ADH	Anxiety and depression scale
12.	AM	Alveolar mucosa
13.	Ant.	Anterior
14.	ARM	Alveolar ridge mucosa;
15.	ATP	Adenosine triphosphate
16.	AT	Apex tongue
17.	BD	Twice a day
18.	BDI	Beck depression inventory
19.	BMS	Burning mouth syndrome
20.	BM	Buccal mucosa
21.	CB1	Cannabinoid receptors type 1
22.	CC4	Complement C4
23.	CCO	Cytochrome c oxidase
24.	CG	Control group
25.	COB	Capillary oral bed
26.	COX-2	Cyclooxygenase- 2
27.	CCRCT	Cochrane Central Register of Controlled Trials
28.	CI	Confidence interval
29.	CITAS	chemotherapy-induced taste alteration scale
30.	CRP	C-reactive protein
31.	cm	Centimetre
32.	cm^2	Square centimetre
33.	CW	Continuous emission mode
34.	DA	Digital algometer
35.	DB	Double-blind
36.	DF	Degrees of freedom
37.	DT	Dorsal tongue

38.	↓	Decrease
39.	et al	And others
40.	ELISA	Enzyme-linked immunosorbent assay
41.	EVT	Entire dorsal tongue
42.	EO	Extraoral
43.	*EuroQol-5D*	European Quality of life-5 dimentions
44.	FM	Floor mouth
45.	F.n.	Fusobacterium nucleatum
46.	GaAlAs	Gallium-aluminium-arsenide laser
47.	GaAs	Gallium-arsenide laser
48.	Ga-Ar	Gallium argo
49.	GMD	Geometric mean diameter
50.	GI	Group I
51.	GII	Group II
52.	GIII	Group III
53.	GIV	Group IV
54.	GV	Group V
55.	G1	Group 1
56.	G2	Group 2
57.	G A	Group A
58.	G B	Group B
59.	G 1	Group 1
60.	G 2	Group 2
61.	GDS	Geriatric depression scale
62.	G	Gingivae
63.	HADS	Hospital anxiety depression scale
64.	HeNe	Helium-neon laser
65.	HP	Hard palate
66.	hr	Hour
67.	↑	Increase
68.	IL	Interleukin
69.	IL-1β	Interleukin-1 beta
70.	IL-1,6,8	Interleukin-1,6,8
71.	IF-γ	Interferon- gamma
72.	IF	Impact Factor
73.	IgA	Immunoglobulin A
74.	IGF-I	Insulin-like growth factor-I
75.	IgG	Immunoglobulin G
76.	IO	Intraoral
77.	IR	Infrared
78.	Inf.	Inferior

79.	InGaAlP	Indium-gallium- aluminum-phosphide laser
80.	IMMPACT II	Initiative on Methods, Measurement, and Pain Assessment in Clinical Trials II
81.	I^2	Statistical test for percentage variation due to heterogeneity
82.	J	Joule
83.	J/cm^2	Joules per square centimeter
84.	KMM	Kaplan-Meier method
85.	LG	Laser group
86.	LEDs	Light emitted diodes
87.	L	Lip
88.	LC-MS-MS	Liquid chromatography with tandem mass spectrometry
89.	LG	Lower gingivae
90.	LLM	Lower labial mucosa
91.	LL	Lower lip
92.	LLLT	Low level laser therapy
93.	LM	Labial mucosa
94.	LM	Lip mucosa
95.	LT	Lateral tongue
96.	NPRS	Numeric Pain Rating Scale
97.	MeSH	Medical Subject Headings
98.	min	Minute
99.	MIP4	Macrophage Inflammatory Protein-4
100.	μm	Micrometer
101.	μ	Micron
102.	μsec	Microsecond
103.	Msec	Millisecond
104.	mm	Millimeter
105.	MMP-1	Matrix metalloproteinase-1
106.	MMP-2	Matrix metalloproteinase-2
107.	MMP-7	Matrix metalloproteinase-7
108.	MMP-9	Matrix metalloproteinase-9
109.	MPQ	McGill Pain Questionnaire
110.	MR	Mandibular ridge
111.	MRI	Magnetic resonance imaging
112.	mW	Milliwatt
113.	M/F	Male/ Female
114.	n	Sample size
115.	N	No
116.	No.	Number
117.	nsec	Nanosecond
118.	nm	Nanometer
119.	NI	No information

120.	NIR	Near infrared
121.	NA	Not applicable
122.	Nd:YAG	Neodymium-doped yttrium aluminum garnet
123.	NS	Not specified
124.	NRS	Numerical rating scale
125.	OS	Oxidative stress
126.	OHIP-14	Oral health impact profile
127.	OHIP-CRO 14	Croatia version of OHIP-14;
128.	P	Palate
129.	PBM	Photobiomodulation
130.	PBMT	Photobiomodulation therapy
131.	PEDF	Pigment epithelium-derived factor
132.	PGE 2	Prostaglandin E2
133.	PG	Parallel group
134.	PGI-I	Patient global impression of improvement
135.	PI	Pain intensity
136.	Post.	Posterior
137.	PPI	Present pain intensity
138.	PPT	Pessure pain threshold
139.	PRISMA	Preferred Reporting Items for Systematic Reviews and Meta-Analyses
140.	PROSPERO	Prospective Register Of Systematic Reviews
141.	PSFS	patient specific functional scale
142.	Q	Cochran's heterogeneity statistic
143.	QoL	Quality of life
144.	ROS	Reactive oxygen species
145.	RoB 2	Revised Cochrane Risk-of-Bias tool for Randomized trials, Version 2.0
146.	RCT	Randomised clinical trial
147.	S	Sublingual
148.	SAP	Serum amyloid P
149.	SCM	Sternocleidomastoid muscle
150.	SCL 90-R	Symptom Checklist-90-R
151.	SD	Standard deviation
152.	SE	Standard error
153.	SB	Single-blind
154.	SF-36	Short Form 36 Health Survey Questionnaire
155.	SOB	Secondary oral burning
156.	SMD	Standardised mean difference
157.	sec/ s	Second
158.	SM	Sublingual mucosa
159.	SP	Soft palate
160.	SSI	Symptoms' severity index

161.	Submand.	Submandibular
162.	Sup.	Superior
163.	T	Tongue
164.	TGF-β1	Transforming growth factor-β1
165.	TNF-α	Tumor necrosis factor- alpha
166.	TNF-β and α	Tumour necrotic factor beta and alpha
167.	TP	Trigger points
168.	TT	Tip tongue
169.	VAS	Visual analogue scale
170.	VM	Vestibular mucosa
171.	VNS	Visual numeric scale
172.	VEGF	Vascular endothelial growth factor
173.	UL	Upper lip
174.	UWS	Unstimulated whole salivary flow;
175.	vs	Versus
176.	W	Watt
177.	W/cm^2	Watts per square centimetre
178.	WBG	Willis Bite Gauge
179.	QoL	Quality of life
180.	QoL-OH	QoL-related Oral health
181.	QST	Quantitative sensory testing
182.	QualST	Qualitative sensory testing
183.	Y	Yes
184.	yrs.	Years
185.	%	Percentage
